# Investigating cement-based surfaces as a sustainable flooring solution to improve *Ascaris Suum* egg removal and inactivation in low-resource settings

**DOI:** 10.1371/journal.pntd.0012919

**Published:** 2025-10-23

**Authors:** Claire E. Anderson, Suhi Hanif, Jason Hernandez, Yoshika Crider, Michael Lepech, Sarah L. Billington, Alexandria B. Boehm, Jade Benjamin-Chung

**Affiliations:** 1 Department of Civil and Environmental Engineering, Stanford University, Stanford, California, United States of America; 2 Department of Epidemiology and Population Health, Stanford University, Stanford, California, United States of America; 3 King Center on Global Development, Stanford University, Stanford, California, United States of America; 4 Chan Zuckerberg Biohub, San Francisco, California, United States of America; The University of Melbourne, AUSTRALIA

## Abstract

Soil-transmitted helminths, like *Ascaris lumbricoides,* are significant contributors to disease burden in low- and middle-income countries (LMICs). Infections are associated with morbidity and mortality in children and are often transmitted through eggs in fecally contaminated soil. Interventions, like replacing household soil floors with cement-based alternatives, may reduce exposure to *A. lumbricoides* eggs, but there are currently no estimates on the removal or survival of *Ascaris* species eggs on cement-based surfaces. This study addresses that knowledge gap by evaluating the removal of *Ascaris suum* eggs from mopping and the survival of *A. suum* eggs on two cement-based mixes: an traditional mortar and a mortar with fly ash, which provides a more sustainable alternative to the traditional mortar mix. We assessed egg survival at two temperatures representing the dry (15°C) and wet (34°C) seasons in Bangladesh using two different egg enumeration methods. After mopping, a mean of 95.6% (SD = 4.0%) of viable eggs were removed from surfaces, with no significant differences between cement-based mixes (p = 0.51). The mean first-order decay rate constants (*k*) of *A. suum* eggs across all conditions was 0.029 day^-1^ (SD = 0.074 day^-1^). Values of *k* were similar between mix designs (p = 0.62) but varied significantly between temperatures (p = 4.2x10^-25^) and egg enumeration methods (p = 2.4x10^-8^). The *k* values were greater at 34°C compared to at 15°C, where they showed no significant inactivation. Our *k* values were comparable to those reported previously for different matrices, indicating comparable inactivation of *Ascaris* species eggs on cement-based surfaces compared to liquid and semi-solid matrices. These results provide some of the first estimates of removal efficiencies and decay rate constants in realistic environmental conditions for *Ascaris* species on surfaces while supporting the use of mortar mix designs with fly ash in interventions to reduce *Ascaris* species transmission in rural LMIC households.

## Author summary

Soil-transmitted helminths, like *Ascaris lumbricoides*, are parasites that are major contributors to disease in children and women of childbearing age in low- and middle-income countries. Interventions, like replacing soil floors in households with cement-based flooring, may reduce exposure to *A. lumbricoides* eggs which cause infections, but there is little information on how or why these interventions may be effective. This study investigates the effectiveness of simple cleaning methods, like mopping, in removing *Ascaris suum* eggs from cement-based surfaces and explores how long these eggs can survive on these surfaces under different environmental conditions. We tested two types of cement-based surfaces, a traditional cement-based mortar mix, and a more sustainable cement-based mortar mix, and found that mopping removed 95.6% (SD = 4.0%) of *A. suum* eggs, with no differences between cement mixes. Experiments simulating wet and dry seasonal conditions showed that *A. suum* eggs survive longer in cooler environments, again with no differences between cement mixes. These findings provide important insights into the role of cement-based flooring in interrupting disease transmission and suggest sustainable cement-based mortar mixes are a feasible alternative to traditional cement-based mortar mixes.

## Introduction

Soil-transmitted helminths infect approximately 24% of the global population, but infections are most common in low- and middle-income countries (LMICs) [1]. Prevalent soil-transmitted helminths include *Ascaris lumbricoides* (roundworm), *Trichuris trichiura* (whipworm), and *Necator americanus* (hookworm) [1,2]. *A. lumbricoides* is a particular concern in Bangladesh, as it is endemic to all 64 districts, and poses the largest risk to preschool-age children, school-age children, and women of childbearing age [3]. In 2010 in Bangladesh, 79.8% of school-age children were infected with one or more helminth species [4]. For *A. lumbricoides*, the most intense infections occur in school-age children and can cause malnutrition, growth stunting, and cognitive deficits [3,5].

Soil floors are common in LMICs [6] and are reservoirs of many pathogens, including soil-transmitted helminths. In studies that collected soil from households to test for soil-transmitted helminths, *A. lumbricoides* eggs were consistently the predominant parasite detected [7,8]. *A. lumbricoides* eggs contaminate soil through the feces of infected individuals, and once eggs are present in soil they can survive for up to 10 years, withstanding extreme weather conditions, and are virtually impossible to remove from soil [2,9]. After fertilized eggs embryonate in the soil, if they are consumed they can infect an individual and continue the pathogen’s life cycle. Children in LMICs are at high risk of exposure to soil-borne pathogens, including soil-transmitted helminths, as they can ingest soil during hand-to-mouth contact [10–15].

Deworming treatments are effective in the short term, but rapid reinfection occurs when environmental reservoirs, such as contaminated soil, are not eliminated. Within six months it is estimated that 68% of those treated become reinfected with *A. lumbricoides* [16]. Ongoing mass school-based drug administration has occurred in Bangladesh since 2008 to reduce *A. lumbricoides* infections, but prevalence is still over 20% in 24 of 64 districts [3,17]. In principle, water, sanitation, and hygiene (WASH) interventions could reduce contamination of soil with *A. lumbricoides* eggs; however, studies investigating the impact of WASH interventions have had inconsistent effects on pathogen removal and disease reduction [8,18–23]. This may be due to a lack of coverage of WASH interventions, inconsistent use of interventions, or because interventions do not sufficiently prevent eggs from reaching soil reservoirs [20,22].

Replacing soil floors with finished flooring, like cement-based floors, may reduce the exposure to soil-transmitted helminths in the home environment by removing a key environmental reservoir. In contrast with WASH interventions, which may require a high level of compliance from communities, intervention uptake is nearly guaranteed with cement-based floors. Observational studies have noted that finished flooring is associated with a lower prevalence of soil-transmitted helminths [24–27], but the mechanisms of disease reduction remain unclear. An ongoing randomized trial in rural Bangladesh, Cement-based flooRs AnD chiLd hEalth (CRADLE, NCT05372068), is currently assessing whether transitioning from soil to cement floors can reduce soil-transmitted helminth infections and diarrhea in children under two years of age [28].

If cement-based floors were to be constructed as a health intervention at scale, they could contribute to significant CO_2_ emissions, as cement production contributes an estimated 5–10% of total global anthropogenic CO_2_ emissions [29,30]. Using an alternative cement mix, such as a mix that replaces a portion of cement with fly ash, would offset CO_2_ emissions for the subset of cement replaced [31–38]; fly ash is a by-product of coal combustion in power plants and is readily available in LMICs like Bangladesh, which rely on coal-fired power generation [39,40]. Additionally, fly ash is a good candidate material to replace cement because it can increase the strength and durability of cement and mortar mixes while offsetting CO_2_ emissions [33,35,37]. Although fly ash may contain heavy metals [41], these metals become largely immobilized in the cement matrix after curing through absorption and chemical reactions [42,43], eliminating significant risks of exposure for household members in contact with fly ash-containing cement-based floors. While replacing a portion of cement with fly ash would offset CO_2_ emissions, it is unknown whether *A. lumbricoides* egg survival and removal would be similar on these surfaces compared to traditional cement-based surfaces. In principle, differences in the cement-based mix’s surface roughness properties, moisture retention, and other factors, could impact the survival and removal of pathogens on each mix’s surface, as pathogens could be shielded from or made more vulnerable to environmental stressors like desiccation. Though similar structural properties have been found between traditional mortar mixes and fly ash mortar mixes [33–38], the impact of fly ash as a cement replacement in mortar mixes on the survival and removal of soil-transmitted helminths is unknown.

When evaluating cement-based flooring as a possible public health intervention to reduce transmission of soil-transmitted helminths like *Ascaris* species, it is important to understand both the persistence of *Ascaris* species on surfaces and the effectiveness of common cleaning methods. While prior studies have investigated the survival of *Ascaris* species in liquid and semi-solid matrices, little to nothing is known about *Ascaris* survival on surfaces. Although research has documented the disinfection potential of both ammonia and 254-nm ultraviolet light on *Ascaris* disinfection in liquid matrices [44–46], it is unclear whether they can remove *Ascaris* species from surfaces. Further, in LMICs, these disinfection products are not attainable for most rural households; instead, common floor cleaning methods include sweeping or mopping with water. The effectiveness of these more common removal methods of *Ascaris* species from surfaces have yet to be assessed.

In this study, we evaluated the removal and survival of *A. suum* eggs on cement-based surfaces while mimicking conditions in a setting where soil-transmitted helminths are common. We also conducted a systematized literature review to compare the survival of *A. suum* eggs on surfaces to additional matrices. We hypothesized that removal and survival would be similar on a typical cement mix and an alternative mix replacing some cement with fly ash. We also hypothesized that *A. suum* first-order decay rate constants would be greater at the wet season temperature, which is warmer than the dry season temperature. The results of this study will indicate if sustainable alternatives to cement-based flooring are appropriate for large-scale infrastructure interventions to prevent the persistence of *Ascaris* species in households and will provide data for use in future predictions of risk of soil-transmitted helminth infections.

## Methods

### Cement-based tiles

Two cement-based mixes were used in these experiments to mimic cement-flooring installed in Bangladesh. The first was a traditional Ordinary Portland Cement (OPC) mortar mix to create cement-based tiles and finished the tiles with a smooth finish of cement paste. The second was an OPC mortar mix with 25% Class F fly ash as a cement replacement to create cement-based tiles and finished those tiles with a smooth cement paste finish containing the same 25% Class F fly ash replacement. In this manuscript, we will refer to the first tile type as the OPC mortar mix tiles and to the second as the OPC fly ash mortar mix tiles. Mix design details are included in the Supplemental Information (Table A in S1 Text).

Cement-based tiles were made according to ASTM C192 Standard Practice for Making and Curing Concrete Test Specimens in the Laboratory [47]. The two cement-based mixes used in this study were prepared in the same manner as those used in Bangladesh in the CRADLE study [28]. The mixes were poured into 127 mm square molds with 12.7 mm depth and allowed to set. After demolding, the tiles were seven-day wet-cured in a lime bath followed by a minimum 28-day air-cure. For testing, each 127 mm square tile was delineated into four, approximately 3 cm x 3 cm, square quadrants using a wax pencil. Some tiles were used twice in experiments; before being used a second time they were disinfected by autoclaving at 121°C and washing thoroughly with water, after which they were allowed to dry at room temperature (approximately 21°C) for a minimum of 5 days before reuse in experiments.

### *A. suum* Stock

*A. suum* was used in these experiments as a surrogate for human pathogenic *A. lumbricoides. A. suum* has been extensively used as a surrogate for *A. lumbricoides* [44,48–52], as the eggs are morphologically and physiologically indistinguishable and their genomes exhibit 98.1% similarity [2,49]. *A. suum* stock was purchased from Excelsior Sentinel, Inc. (Trumansburg, NY, USA). The stock solution was made up of roughly 10^6^ fertilized *A. suum* eggs, derived from sieved pig feces, and 50 mL of 0.1 N sulfuric acid to prevent mold growth. Upon arrival, the egg stock solution was stored at 4°C to prevent eggs from embryonating; eggs were stored between two and seven months before use in experiments. The stock solution was monitored throughout the storage time period to ensure the eggs within the stock did not develop over time and to estimate the number of fertilized eggs at the start of the experiments. The proportion of fertilized eggs in the stock was determined by spiking 25 μL of stock solution to 15 mL of autoclaved deionized (DI) water and 15 mL of 0.1 N sulfuric acid (Sigma-Aldrich, St. Louis, MO, USA). Stock egg enumeration methods were the same as experimental sample enumeration, and are described in a subsequent Methods subsection. Each time prior to using the stock, the solution was inverted 10 times to ensure a well-mixed solution.

The dry weight of the total solids in the stock solution was measured to estimate the amount of organic and particulate material present on the cement surfaces. The weight was determined by spiking 25 μL of the stock solution onto a weigh boat and allowing the sample to dry for approximately three hours, until the stock was no longer visibly wet. The average recorded weight after drying for five samples was used to determine the dry weight of the total solids per volume of the stock.

### Outcomes

The study outcomes included 1) the percent removal of viable eggs on the two different cement-based mixes from mopping, 2) the first-order decay rate constants of the eggs (both viable eggs and the broader category of developed eggs) over time on the two cement-based mixes in high- and low-temperature conditions, and (3) a review of present literature to assess how the first-order decay rate constants of the eggs on cement surfaces compares to other matrices.

### Experimental procedure

Experiments took place at Stanford University between December 2023 and July 2024.

#### Removal experiments.

These experiments simulate *A. suum* eggs on two different types of cement-based floors before and after mopping. On a single cement-based mix tile, each of the four quadrants on the tile was inoculated with *A. suum* eggs as described later in the Methods. After inoculation, two of the quadrants were designated as no-mopping controls and the *A. suum* was recovered and enumerated (detailed in subsequent methods). The remaining two quadrants were mopped with two damp 16 cm^2^ 100% cotton cloths (Nabob Wipers, Brooklyn, NY, USA), wet with approximately 5 mL of autoclaved DI water. The two quadrants were wiped twice with side-to-side motions until the entire square was visibly damp. The remaining *A. suum* were then recovered from the tile. Each no-mopping control quadrant and mopping quadrant pair on the tile represents one trial. Twenty-six (26) trials were performed on tiles of each of the two cement mixes (26 trials x 2 mix design groups = 52 trials total). This n was chosen for the removal and survival experiments to provide 80% power for the main outcomes of the study. The experiments were powered to measure large effect sizes according to a unitless, standardized measure of effect size for each statistical test used [53,54]. To determine differences in the percent removal of eggs between tiles of the two cement mixes, a minimum sample size of 26 per group was required for an unpaired, two-tailed t-test.

#### Survival experiments.

These experiments simulate *A. suum* egg survival on cement-based flooring in LMICs like Bangladesh. Specifically, we evaluated survival in the wet season of Bangladesh (34°C, 75% relative humidity (RH)) and dry season (15°C, 75% RH). The wet season typically ranges from April to October in Bangladesh. In Tangail, a city near the CRADLE study site, the monthly average of daily high temperatures peaked in April, at 33.9°C, and the relative humidity was 74% [55,56]. The dry season typically ranges from November to February, and in Tanagail the lowest mean monthly minimum temperature (in January) was 11.4°C and the relative humidity was 80% [55,56]. Relative humidity was kept constant at 75% in this study through sealed containers with saturated salt solutions [57] and temperature was held constant at either 15°C or 34°C through incubators. A single cement tile represented one trial and was split into four quadrants for each of the 4 time points studied (0, 2, 14, and 28 days). Sampling time points were chosen based on previous *Ascaris* species viability [45,58–60]. Given the range of temperatures tested in our study and the novelty of examining *Ascaris* species survival on surfaces, we opted for a more conservative sampling timeline spanning days to weeks. This approach ensured that sampling occurred before complete inactivation had taken place at most time points, allowing us to better characterize decay over time. Additionally, because we anticipated a log-linear decay pattern consistent with Chick’s Law, we included more frequent sampling at earlier time points.

All four quadrants were inoculated using the procedure described in the subsequent Methods subsection. After inoculation, samples were sacrificially sampled at each time point, meaning that the sample was removed from the tile and was not placed back for future time points. Instead, another quadrant was sampled for later time points. Thirteen (13) trials were conducted for each cement mix at two season temperatures, the wet season and dry season (13 trials x 2 mix designs x 2 temperatures = 52 trials total). To determine differences in the first-order decay rate constants of the eggs between the two cement mixes and two temperature conditions with 80% power, a minimum sample size of 13 per group was required for an Analysis of Variance (ANOVA) with fixed effects.

#### Inoculation.

Cement tiles were inoculated with *A. suum* prior to removal and survival experiments. To inoculate the cement tiles with *A. suum*, 25 μL of the stock solution (containing *A. suum* eggs, sieved pig feces containing both organic and particulate matter, and 0.1 N sulfuric acid) was diluted in 2 mL of autoclaved DI water. The solution was spread evenly across one 9 cm^2^ quadrant of the tile using a sterile disposable plastic needle (Fisherbrand, Waltham, MA, USA) and allowed to dry for approximately 1 hour until the surface was no longer visibly wet. After drying, the removal experiments were performed or the tile was subject to additional incubation for the survival experiments, as described in those Method sections.

#### Recovery.

Eggs were recovered from the cement tile using a new method, referred to as the “washing method” in this study. With the washing method, roughly 3 mL of autoclaved DI water was added to one 9 cm^2^ quadrant of the tile, and the surface of the quadrant was agitated with a sterile disposable plastic needle (Fisherbrand, Waltham, MA, USA). The water was pipetted off the surface and added to a sterile, 50 mL centrifuge tube (CORNING, Corning, NY, USA). The process of water addition, agitation, and recovery was repeated until 15 mL of water was collected from the tile quadrant; this typically took 10–15 minutes to achieve per quadrant of the tile. Then, 15 mL of 0.1 N sulfuric acid (Sigma-Aldrich, St. Louis, MO, USA) was added to the tube to prevent mold growth, resulting in a total volume of 30 mL per sample. To embryonate the eggs, the sample was incubated in darkness for 32–35 days at 26°C. This combination of incubation time and temperature is sufficient to embryonate the maximum number of eggs in the sample [48,61,62].

#### Enumeration.

*A. suum* eggs were enumerated using microscopy. Samples were centrifuged at 1000 x g for three minutes and 29 mL of the supernatant was removed and discarded according to biosafety guidelines. The remaining 1 mL sample was transferred to a Sedgewick-Rafter slide (Electron Microscopy Sciences, Hatfield, PA, USA) for enumeration under a microscope [63]. A microscope (Swift Optical Instruments, Inc. San Antonio, TX, USA) was used to view the slide at 10X magnification in the program ToupLight (ToupTek, Hangzhou, Zhejiang, P.R. China) with a 0.5X magnification camera attachment (OMAX Microscopes, Irvine, California, USA).

Eggs were counted and classified into one of 16 development stages [62] or as dead/non-viable. The total number of eggs includes all development stages, dead, and non-viable eggs. We determined viability through both conventional and developmental methods [62]. For the conventional enumeration method, eggs at stage 15 (which had the most well-developed larvae prior to excystation) of the developmental process were considered viable and all other eggs were considered non-viable. For the developmental enumeration method, we grouped into five categories based on their development stages: (1) Unembryonated, stage 1; (2) Embryonated, stage 2–7; (3) Well-developed, stage 8–15; (4) Excystation, stage 16; and (5) Dead or non-viable. Example photos of development categories are shown in the Supplemental Information (Fig A in S1 Text).

Viable *A. suum* eggs are conventionally defined as embryonated eggs containing mobile, distinguishable larvae. Fertilized eggs become viable if they are incubated under the right conditions, while dead or unfertilized eggs cannot become viable. In conventional microscopy methods, all eggs without larvae are considered non-viable [48,62,64], however, this may undercount the total number of potentially viable eggs. For example, eggs that have larvae in well-developed stages may still be capable of later development to become fully viable. Thus, previous studies have reasoned that eggs containing mobile, distinguishable larvae as well as eggs with well-developed larvae should be considered when determining viability [48,62,64]. For removal experiments, we quantified viable eggs as eggs at developmental stage 15 (conventional method). For survival experiments, we quantified viable eggs in two ways, (1) as eggs at developmental stage 15 (conventional method) and (2) as eggs in the well-developed, third category of stages (developmental method).

#### Controls.

Positive and negative controls were included to assess the number of eggs which were applied to and recovered from cement-based tiles. Positive *A. suum* egg application controls were created by spiking 0.25 μL *A. suum* stock directly into a 50 mL centrifuge tube with 15 mL water. Then, 15 mL of 0.1N sulfuric acid was added, and the sample was incubated in darkness for 32–35 days at 26°C and the number of total and viable eggs was enumerated. Positive *A. suum* application controls provide insight into the total number of eggs and the number of viable eggs applied to the tile. Positive *A. suum* recovery controls from the cement-based tiles were created by inoculating tiles as previously described and then recovering the sample and incubating in darkness for 32–35 days at 26°C and enumerating the total number of eggs recovered. Positive *A. suum* recovery controls were sampled immediately after application without the mopping procedure for the removal experiments. For survival experiments, positive *A. suum* recovery controls were sampled immediately after application (time = 0 days), and at each time point (2, 14, and 28 days) for the 34°C condition, 15°C condition, OPC mortar mix tiles, and OPC fly ash mortar mix tiles. Positive *Ascaris* recovery controls give insight into the total number of *A. suum* eggs recovered from the tile surface after initial application given our washing method. The positive *A. suum* recovery controls differ from the main study outcome because they measure the total number of eggs recovered, versus the number of viable or developed eggs. Negative controls were created by choosing a random quadrant on each cement mix tile prior to *A. suum* inoculation. 2 mL of autoclaved DI water was added to one 9 cm^2^ quadrant of the tile and allowed to dry until the surface was no longer visibly wet (~1 hour). The negative control was then recovered using the washing method and incubated in darkness for 32–35 days at 26°C. The positive and negative controls were enumerated as previously described.

The number of control samples collected varies based on the sample type collected. The positive controls collected can be divided into two categories: application controls and recovery controls. Sixty-six (66) application controls were measured in total for the removal and survival experiments. Two-hundred and sixty (260) recovery controls were measured in total for the removal experiments; 52 for the removal conditions (26 trials x 2 cement mixes) and 208 for the survival conditions (13 trials x 4 time points x 2 temperature conditions x 2 cement mixes). Eighty (80) tiles were used throughout the experiments, and negative controls were measured for each tile.

### Data analysis

Data analysis was performed with R (R: A Language for Statistical Computing, version 1.2.5042; R Foundation for Statistical Computing, Vienna, Austria) and G*Power [53].

The percent removal of *A. suum* eggs was calculated using Equation 1:


$$Percent\;Removed\;=\;(\text{1}-\frac{N}{N_{\text{0}}})*\text{100}\%$$ Equation 1

where *N* is the number of viable eggs after the mopping and *N*_*o*_ is the initial number of viable eggs before mopping. The number of viable eggs for removal experiments was determined through the conventional method of viability assessment.

We assessed survival using percent viability and calculation of first-order decay rate constants. Calculations used both the number of viable eggs through the conventional method and the developmental evaluation method (referred to as “Developed” in the survival experiment results). We used Equation 2 to calculate percent viability:


$$Percent\;Viable=\frac{N_{V}}{N_{T}}*\text{1}\text{0}\text{0}\%$$ Equation 2

where *N*_*V*_ is the number of viable at each time point and *N*_*T*_ is the total number of eggs recovered at each time point.

To estimate the first-order decay rate constant (*k*), we fit the first-order log-linear regression with a normal family and identity link described in Equation 3:


$$ln(\frac{N_{V}}{N_{\text{0}}})=-kt+\beta_{\text{0}}$$ Equation 3

where *N*_*o*_ is the initial number of viable eggs, *N*_*V*_ is the number of viable eggs after a time of *t* (days) on the cement-based surface, *k* is the first-order decay rate constant (days^*−*1^), and β_0_ is the y-intercept. While some studies use log reductions with a lag [65,66], we did not observe a lag phase in our data (Fig B in S1 Text). Models were fit for each experimental condition (4 regressions, 2 mix designs x 2 temperatures) as well as for each trial to obtain *k* for statistical analysis.

An unpaired, two-tailed t-test was used to determine differences in removal experiment results, while an ANOVA was used for differences in the *k* of the survival experiments. Interactions were not considered in our analysis. A Tukey honestly significant post-hoc test followed the ANOVA. Additional unpaired, two-tailed t-tests and ANOVAs with fixed effects were performed on subsets of data to determine differences in controls and variances over time. To explore potential effects of surface roughness and temperature on egg recovery, we conducted an ANOVA to test the influence of tile type, time, and environmental conditions. Data was tested for normality using Shapiro-Wilk tests and all analyses were performed using a significance level of *α *= 0.05.

### Literature review of *Ascaris* species survival at different temperatures

Our goal with this literature review was to extract from the peer-reviewed literature the time to 99% inactivation data of *Ascaris* species under a wide range of conditions and in/on a variety of matrices. Our aim was to compile these data for comparison to the data collected herein. We conducted a systematized literature review of peer-reviewed articles broadly related to *Ascaris* species survival in temperature conditions relevant to human habitats. A systematized literature review is similar to, but less stringent than, a systematic review [67].

The literature review included articles published up until December 16, 2024 using Web of Science, PubMed, Scopus, and Google Scholar. We used the search string (survival OR decay OR inactivation OR fate OR persistence OR viability) AND (“ascaris” OR roundworm) AND (temperature*) across titles, abstracts, and keywords in Web of Science, PubMed, and Scopus and incorporated all articles found. We used the search phrase “effect of temperature on ascaris” in Google Scholar and incorporated the first 50 articles generated. After duplicates were identified in Covidence (Covidence, Melbourne, Australia) and removed, 199 articles remained. Articles were then screened by one reviewer for the following requirements: (1) publication in English, (2) primary data collection (no reviews or modeling papers), (3) microscopy experiments with *Ascaris* species eggs, (4) constant-temperatures experiments, (5) experimental (or control) temperatures less than 45°C, (6) experiments (or controls) without inactivation methods besides temperature, and (7) decay-rate constants, inactivation time, or reduction in viable *Ascaris* species eggs reported.

We chose to include studies that investigated inactivation at temperatures less than 45°C because 45°C is at the upper bound of temperatures in which humans habitate; additionally, at temperatures below 45°C, *Ascaris* species eggs are thought to have a different inactivation-temperature relationship than higher temperatures [59,65,68]. We also only included data from studies with low ammonia (no added ammonia and no studies in only urine) and where pH was typically neutral, as ammonia has been found to promote the inactivation of *Ascaris* species eggs and pH has had mixed effects [46,51,66,69–71]. Additionally, we only included results from aerobic studies, as floors in households are part of an aerobic environment and most studies agree that aerobic conditions accelerate inactivation [59,65].

After title/abstract screening of the initial 199 articles, 83 articles remained for full-text screening. After full-text screening, 26 papers were selected for data extraction. For five studies, data was approximated from figures using PlotDigitizer (PORBITAL). Time to 99% inactivation, when not reported by the study, was calculated assuming a log-linear inactivation model, even if a lag time was reported.

## Results

### Removal experiments

**Fig 1:**
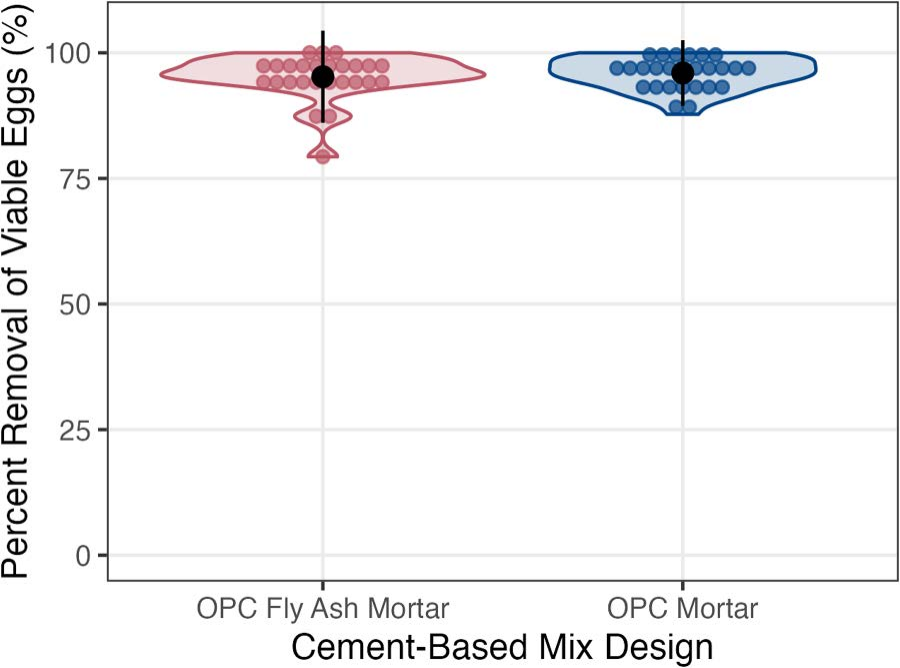
Percentage removal of viable eggs OPC fly ash mortar and OPC mortar cement-based tiles. All 26 data points for each condition are shown in a dot plot, overlaid by a density plot where the width indicates frequency. The black dot indicates the mean removal percentage and the error bars show the mean ± the standard deviation.

The mean removal of viable eggs by mopping for OPC fly ash mortar mix tiles was 95.2% (SD = 4.6%) and 95.9% (SD = 3.3%) for OPC mortar mix tiles (Fig 1). A t-test comparing OPC fly ash mortar mix tiles and OPC mortar mix tiles showed no significant difference in the removal efficiency of viable eggs by mopping between the groups (p = 0.51) (Fig 1). Similarly, tile type did not significantly impact total *A. suum* egg recovery for the removal experiments (p = 0.10).

### Survival experiments

**Fig 2:**
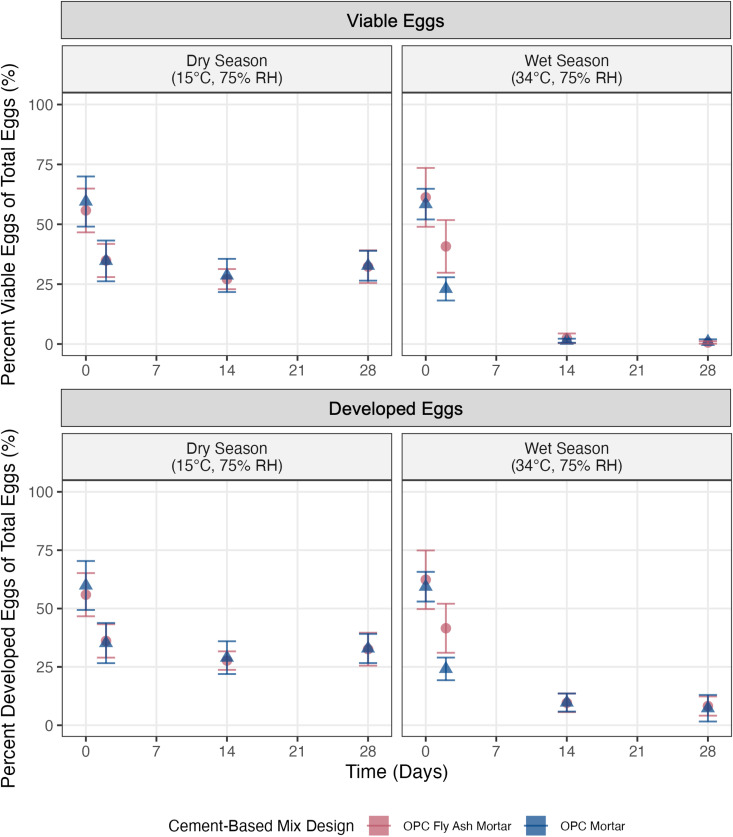
Graph of the mean percent viable eggs and percent developed eggs at time 0, 2, 14, and 28 days. Thirteen trials were averaged for each environmental condition (wet season, 34° and 75% RH; dry season, 15°C and 75% RH) and cement-based mix design (OPC fly ash mortar and OPC mortar). The points and error bars show the mean ± the standard deviation.

The percent of viable eggs of total eggs (Equation 2) did not differ between the OPC fly ash mortar mix tiles and the OPC mortar mix tiles at the 0, 14, and 28-day time points (Fig 2, individual trial graphs available in the SI) for either enumeration method used. In contrast, at two days, percentages from both enumeration methods differed by mix design (p = 2.4 x 10^-3^ and p = 6.2 x 10^-4^, respectively). When the data were analyzed over the total length of the experiment rather than the individual time points, the *k* values from the linear regressions (Equation 3) of *A. suum* egg survival for each trial (Figs B, F and G in S1 Text) were similar between mix designs (p = 0.62). Across all survival experiments, the median *k* value was 0.029 day^-1^.

The *k* values (Table 1) varied significantly between temperatures (p = 4.2 x 10^-25^) and enumeration methods (p = 2.4 x 10^-8^). At 34°C, when all trials were combined, *k* values were 0.15 day^-1^ (p = 2.3 x 10^-20^) and 0.060 day^-1^ (p = 1.1 x 10^-11^) for conventional and developmental enumeration, respectively, on fly ash mortar tiles and 0.13 day^-1^ (p = 2.9 x 10^-17^) and 0.066 day^-1^ (p = 1.1 x 10^-12^) for conventional and developmental enumeration, respectively, on mortar tiles. These values indicate the inactivation of *A. suum* eggs with time, as *k* values are significantly different from zero. In contrast, at 15°C, no significant differences in *k* from zero were observed. At 15°C, when all trials were combined, *k* values were -1.1 x 10^-3^ day^-1^ (p = 0.82) and -7.9 x 10^-4^ day^-1^ (p = 0.87) for conventional and developmental enumeration, respectively, on fly ash mortar tiles and 1.4 x 10^-3^ day^-1^ (p = 0.80) and 1.5 x 10^-3^ day^-1^ (p = 0.78) for conventional and developmental enumeration, respectively, on mortar tiles. After 28 days, there was a mean reduction of eggs quantified using the conventional enumeration method of 59.2% at 34°C and 25.5% at 15°C, across trials and cement-based mix designs. Post-hoc tests confirm that wet season, higher temperature *k* values exceed dry season, low-temperature *k* values (p = 1.2 x 10^-10^, mean difference of 0.11 day^-1^ in *k* between conditions). Post-hoc tests also confirm that *k* values obtained using the conventional method counts had a greater magnitude (indicating faster inactivation in the same time period) than those obtained from developed egg counts (p = 2.3 x 10^-8^, mean difference of 0.05 day^-1^ in *k* between enumeration methods).

**Table 1:** Regression results, including first-order decay rate constants (*k*) and y-intercept values (β_0_) for each cement-based mix design, experimental condition, and enumeration method used.

Mix Design	Environmental Condition	Enumeration Method	*k* (day^1^)	*k* p-value	*β*_0_	*β*_0_ p-value	R^2^
OPC Fly Ash Mortar	Wet Season (34°C, 75% RH)	Viable	0.15	2.3 x 10^-20^	-0.24	0.10	0.83
		Developed	0.060	1.1 x 10^-11^	-0.23	0.03	0.61
	Dry Season (15°C, 75% RH)	Viable	-1.1 x 10^–3*^	0.82	-0.14	0.07	1.0 x 10^–3^
		Developed	-7.9 x 10^–4*^	0.87	-0.12	0.10	5.6 x 10^–4^
OPC Mortar	Wet Season (34°C, 75% RH)	Viable	0.13	2.9 x 10^-17^	-0.32	0.03	0.81
		Developed	0.066	1.1 x 10^-12^	-0.21	0.07	0.63
	Dry Season (15°C, 75% RH)	Viable	1.4 x 10^–3*^	0.80	-0.10	0.25	1.3 x 10^–3^
		Developed	1.5 x 10^–3*^	0.78	-0.09	0.27	1.2 x 10^–3^

* P-values for *k* indicate that the first-order decay rate constant is not significantly different from zero, and that therefore no inactivation was observed.

The average recovery of *A. suum* eggs from the tiles across all conditions was 34% of the total applied eggs. Tile type did not significantly impact the recovery of total *A. suum* eggs from the tiles (p = 0.17), but both time (p = 5.7 × 10 ⁻ ⁹) and temperature (p = 1.2 × 10 ⁻ ⁵) significantly influenced recovery in survival experiments. Overall, recovery increased from an average of 128 eggs at time 0 (25% of the total applied eggs) to 202 eggs at 28 days (40% of the total applied eggs). At the low temperature, an average of 156 eggs were recovered (31% of the total applied eggs), whereas in the high temperature, the mean recovery was 194 eggs (38% of the total applied eggs) (Fig D in S1 Text). We have included all recovery percentages, as well as a correction for *k* for recovery differences over time, in the SI. The correction resulted in slightly increased k values (mean difference of 0.014 day^-1^; median *k* value of 0.042 day^-1^ with the correction versus 0.029 day^-1^ using unadjusted data). In the main text, we report unadjusted decay-rate constants as a conservative estimate of *A. suum* egg inactivation.

### Controls

We did not detect any *A. suum* eggs in our negative controls (samples recovered from the cement-based tiles before *A. suum* inoculation). For positive controls, the pre-incubation controls showed that over 98% of eggs were fertilized (single-celled) in the stock solution from 4°C storage. The application controls showed that 95.9% (SD = 1.9%) of eggs successfully larvated in the positive controls after incubation for 32–35 days at 26°C. Additionally, the mean number of total eggs applied to the cement-based tiles across experimental conditions was 510 eggs (standard deviation, SD = 106 eggs) across all trials and this was similar between mix designs (p = 0.87). The distribution of the total number of eggs applied to the tiles was normal, as confirmed by the Shapiro-Wilk test (p = 0.60) and visualized in a histogram (Fig C in S1 Text). In terms of recovery controls, the mean number of total eggs recovered from the tiles was 172 eggs (34% of applied eggs, SD = 65 eggs) and the number of total eggs recovered had a normal distribution (Shapiro-Wilk test p = 0.60).

### Experimental Setup

The dry weight of the solids in the stock volume controls yielded a mean dry weight of 3.26 mg for 0.25 µL of stock solution, yielding approximately 3.6 g/m² of solids in the experiments when spread over the tile quadrant. This concentration reflects the total organic and particulate matter present on the cement surfaces, which could influence *A. suum* egg survival and removal.

### Literature Review of *Ascaris* Species Survival at Different Temperatures

**Fig 3:**
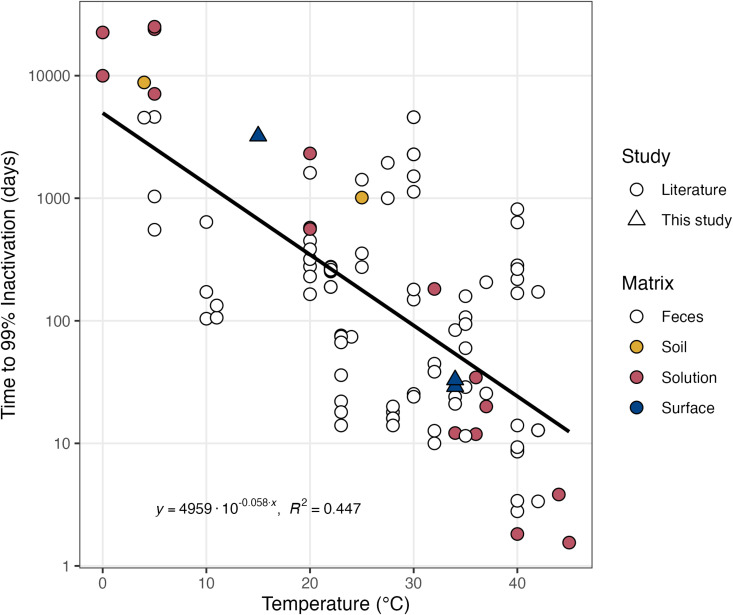
Time to 99% inactivation of *Ascaris* species eggs at different temperatures as a result of the literature review. Data point shape indicates the origin of the data and color represents the matrix studied. A regression of the data, along with the equation and R2, are also plotted. Complete references and study information are described in the SI.

The literature review revealed wide variability in egg survival time across diverse matrices, with temperature as an important factor influencing time to 99% inactivation. Across 27 studies (including this study), the most common matrix for measuring *Ascaris* species egg survival was fecal sludge and only this study provided survival estimates on solid surfaces [45,46,58–60,65,66,72–90]. Matrices and study types were simplified for visualization in Fig 3. The complete data set and visualization are available in the SI. Temperatures investigated ranged from 0 to 44°C, and 105 unique data points of time to 99% inactivation were reported or derived from literature data. Most (23/26) studies from the literature used the conventional method of enumeration. To compare our results to these studies, we used the results of our study obtained with the conventional method of enumeration. In our study, to achieve 99% inactivation of *Ascaris* species eggs on cement-based surfaces, an inactivation time of 29–33 days is needed for surfaces at an ambient temperature of 34°C. In contrast, over 3000 days (8.8 years) is required to achieve 99% inactivation of *Ascaris* species eggs on cement-based surfaces at 15°C.

Generally throughout the studies, at higher temperatures, the time to 99% inactivation of *Ascaris* species eggs was lower. This relationship was observed across all matrices. From the literature data set and the data obtained in this study, we approximated the time-temperature relationship using a linear regression of temperature and log_10_-transformed time values (Equation 4). The resulting equation for *Ascaris* species egg inactivation at temperatures relevant to human environmental exposures is:


$$t_{\text{9}\text{9}}=\text{4}\text{9}\text{5}\text{8}*\text{1}{\text{0}}^{-\text{0}.\text{0}\text{5}\text{8}\;*\;T}$$ Equation 4

where *t*_*99*_ is the time for 99% inactivation in days and *T* is the temperature in °C. This regression has an R^2^ value of 0.45. Using Equation 3, we estimate a general time of 99% inactivation of *Ascaris* species eggs of 669 days (1.8 years) at 15°C and 53 days at 34°C.

## Discussion

We found that *A. suum* survival and removal from cement surfaces were similar between different cement mixes, including a mix with lower embodied carbon. These findings support the use of these mixes in the ongoing randomized control trials to measure health impacts. The study outcomes indicate that mopping is a viable cleaning method for *A. suum* and that the time to inactivation is longer at cooler temperatures, suggesting that cooler temperatures may be an important condition to consider when planning public health interventions to reduce transmission of soil transmitted helminths.

The mean removal percentage of *A. suum* eggs from mopping was 95.6% (SD = 4.0%) across cement-based mix designs tested. Overall, the removal percentage was lower than reported in prior studies, which investigated removal in liquid matrices, with removal methods including adding ammonia, using 254-nm ultraviolet light, and filtering [44–46]. While these experiments often observed high inactivation or removal rates of *A. suum* eggs (>99% under some conditions), the results of these studies are not directly applicable to the removal of *A. suum* from surfaces, especially in LMICs, where many chemical products are not attainable for rural households. Thus, while the removal efficiency from mopping (>92%) is lower than that achieved with chemical disinfectants, it is more applicable to low-income, rural settings where access to chemical products is limited.

In terms of *A. suum* egg survival on cement-based surfaces, our results showed that *k* did not differ by cement mix design, but were influenced by temperature. Specifically, at 34°C, *k* had a greater magnitude, indicating faster inactivation, than at 15°C. Moreover, *k* at 15°C was not significantly different from zero, indicating no inactivation was observed during the dry season conditions. A low *k* at low temperatures for human habitats agrees with previous studies, which found that the *k* values of *A. suum* eggs largely depended on temperature and oxygen availability. In previous studies, the *k* values increased in magnitude with temperature at a predictable rate, and at temperatures above 60°C, eggs were inactivated within a few minutes [68,91].

Our findings are consistent with *Ascaris* species survival trends in different environmental matrices reported in other studies. When comparing our results to studies selected from our literature review with inactivation rates in realistic environmental conditions (<45°C), results suggest that the time to 99% inactivation of *Ascaris* eggs on cement-based surfaces is similar to liquid and semi-solid matrices, especially at 34°C (Fig 3 and S1 Text). Similar inactivation times of *Ascaris* species eggs between cement-based surfaces and liquid and semi-solid matrices indicate that replacing soil floors with cement-based flooring may not inherently reduce *Ascaris* species egg concentrations, as there is no increased inactivation on the cement-based surface. Instead, decreased *Ascaris* species egg concentrations on cement-based surfaces may be a result of the ease of cleaning the surface, as discussed in the mopping experiments.

The *k* values reported for *A. suum* eggs in this study may be impacted by the high concentration of total solids present in the spike solution (3.6 g/m²), which exceeds typical field levels reported in rural Bangladeshi households with cement-based floors (0.2 g/m², SD = 0.3, of dust weight from sweeping) [92]. The solids likely consist mostly of organic matter, and organic matter like fecal matter has been shown to protect *Ascaris* eggs from environmental stressors [65], suggesting that our experimental conditions may have overestimated egg survival compared to typical household scenarios. Additionally, some previous studies have observed a lag phase of up to 12 weeks [65,66] in the inactivation of *A. suum,* which we may have observed under the conditions described herein if we sampled for a longer time.

The choice of enumeration method significantly affected inactivation estimates; conventional enumeration yielded larger *k* values compared to the developmental enumeration method. This discrepancy indicates that conventional methods may underestimate viable egg numbers by excluding late developmental stages and is in line with hypotheses from previous studies [48,62,64]. The resultant difference depended on the temperature of the survival experiments. In higher temperature conditions, the difference between the *k* values calculated from conventional and developmental enumeration methods was greater. Additionally, at the higher temperature, the linear decay model has increased R^2^ values, indicating it fits better for the conventional enumeration versus the developmental enumeration method. These results emphasize that while the conventional method of enumeration simplifies decay modeling, it may provide an incomplete picture of *A. suum* egg survival.

The recovery percentage of *A. suum* eggs from cement-based surfaces in this study was approximately 34% and did not vary by cement mix, although variations were observed across all samples under different temperatures and times. Changes in recovery based on temperature and time are likely due to changes in egg morphology, such as desiccation or alterations in surface adhesion properties. Recovery differences over time could have implications for survival and removal scenarios in the field. For instance, eggs from fresh fecal contamination on cement-based flooring may be less easily removed from cement-based surfaces. With time, dried eggs may become less adherent to the cement-based surfaces, facilitating their removal by mopping but also increasing their potential for dispersal and transmission.

A limitation of this study is that while mopping removed a significant proportion of *A. suum* eggs from cement-based tile surfaces, the eggs may not have been inactivated. Viable eggs may persist on cleaning tools, such as mops, which could lead to cross-contamination. This phenomenon was observed in one previous study which investigated the disinfection of glass and plastic surfaces and found that while eggs were removed from surfaces, they were not inactivated on the cleaning tool [93]. Although additional passes with a cleaning tool could potentially enhance removal efficiency, additional passes may also increase the likelihood of eggs adhering to the cleaning tool and being redistributed elsewhere within the household. Future studies could investigate *A. suum* egg survival on cleaning tools, redistribution of eggs elsewhere in the home, and potential disinfection methods for cleaning tools. Additional limitations relate to the field-applicability of these experiments, which were performed in a lab setting. Although *A. suum* is very similar to *A. lumbricoides*, and is a commonly used surrogate in laboratory experiments, it may behave differently than the human pathogen or other soil-transmitted helminths. Additionally in the field, *A. lumbricoides* eggs may be subject to additional stressors or removal methods. For example, households may use sweeping instead of mopping, and transport throughout households on shoes or feet may put mechanical stress on the eggs, reducing their viability.

Overall, this study contributes novel insights into both *Ascaris* species egg removal and survival dynamics on surfaces, providing some of the first estimates of removal and *k*. These results can inform future research by providing key parameters for modeling *Ascaris* species persistence on surfaces under different environmental conditions. Specifically, results can be incorporated into quantitative microbial risk assessments to estimate infection risks and evaluate the potential impact of health-related flooring interventions. Such analyses can offer evidence of intervention effectiveness to stakeholders, including funders, supporting public health programs. Repeated trials (26 per mix design for removal experiments and 13 per condition for survival experiments) strengthened our results. Results indicate that cement-based flooring may reduce *Ascaris* egg concentrations due to easier cleaning methods, but survival was similar to other matrices. By conducting experiments with both a traditional and a more sustainable cement-based flooring option, our results show that *A. suum* eggs behaved similarly on both surfaces in terms of removal and survival. Our results highlight that the sustainable OPC fly ash mortar mixes can serve as an effective alternative to traditional cement-based flooring in disease interventions, as both have potential strengths to reduce *Ascaris* species transmission in rural LMIC households.

## Supporting information

S1 Text Table A: Cement-based tiles mix designs. Table B: Recovery percentages for experimental conditions. Table C: Regression results corrected and unadjusted data. Fig A: *Ascaris suum* egg development photos. Fig B: Natural Log reduction of *Ascaris suum* viable eggs by trial. Fig C: Number of *Ascaris suum* eggs applied. Fig D: Number of *Ascaris suum* eggs recovered. Fig E: Natural log reduction of eggs for corrected and unadjusted data. Fig F: Survival experiments *Ascaris suum* viable eggs by trial. Fig H: Survival experiments *Ascaris suum* developed eggs by trial. Fig G: Literature review of time to 99% inactivation of *Ascaris* species eggs.
(DOCX)

## References

[1] World Health Organization. Soil-transmitted helminth infections. In: Fact sheets. 18 Jan 2023 cited 24 Sep 2024. https://www.who.int/news-room/fact-sheets/detail/soil-transmitted-helminth-infections

[2] Dold C, Holland CV. Ascaris and ascariasis. Microbes Infect. 2011;13(7):632–7. doi: 10.1016/j.micinf.2010.09.012 20934531

[3] Nath TC, Padmawati RS, Alam MS, Das S, Murhandarwati EH. Elimination of soil-transmitted helminthiasis infection in Bangladesh: Knowledge, attitudes, and practices regarding mass drug administration. Journal of Global Health Reports. 2018;2. doi: 10.29392/joghr.2.e2018017

[4] Hafiz I, Berhan M, Keller A, Haq R, Chesnaye N, Koporc K, et al. School-based mass distributions of mebendazole to control soil-transmitted helminthiasis in the Munshiganj and Lakshmipur districts of Bangladesh: an evaluation of the treatment monitoring process and knowledge, attitudes, and practices of the population. Acta Trop. 2015;141(Pt B):385–90. doi: 10.1016/j.actatropica.2013.12.010 24370675

[5] Bethony J, Brooker S, Albonico M, Geiger SM, Loukas A, Diemert D, et al. Soil-transmitted helminth infections: ascariasis, trichuriasis, and hookworm. Lancet. 2006;367(9521):1521–32. doi: 10.1016/S0140-6736(06)68653-4 16679166

[6] Colston JM, Fang B, Nong MK, Chernyavskiy P, Annapareddy N, Lakshmi V, et al. Spatial variation in housing construction material in low- and middle-income countries: A Bayesian spatial prediction model of a key infectious diseases risk factor and social determinant of health. PLOS Glob Public Health. 2024;4(12):e0003338. doi: 10.1371/journal.pgph.0003338 39693286 PMC11654929

[7] Steinbaum L, Njenga SM, Kihara J, Boehm AB, Davis J, Null C, et al. Soil-Transmitted Helminth Eggs Are Present in Soil at Multiple Locations within Households in Rural Kenya. PLoS One. 2016;11(6):e0157780. doi: 10.1371/journal.pone.0157780 27341102 PMC4920396

[8] Steinbaum L, Mboya J, Mahoney R, Njenga SM, Null C, Pickering AJ. Effect of a sanitation intervention on soil-transmitted helminth prevalence and concentration in household soil: A cluster-randomized controlled trial and risk factor analysis. PLoS Negl Trop Dis. 2019;13(2):e0007180. doi: 10.1371/journal.pntd.0007180 30742614 PMC6386409

[9] CDC (Centers for Disease Control and Prevention). CDC - Ascariasis - Prevention & Control. 11 Apr 2019 https://www.cdc.gov/parasites/ascariasis/prevent.html

[10] Julian TR. Environmental transmission of diarrheal pathogens in low and middle income countries. Environ Sci Process Impacts. 2016;18(8):944–55. doi: 10.1039/c6em00222f 27384220

[11] Morita T, Perin J, Oldja L, Biswas S, Sack RB, Ahmed S, et al. Mouthing of Soil Contaminated Objects is Associated with Environmental Enteropathy in Young Children. Trop Med Int Health. 2017;22(6):670–8. doi: 10.1111/tmi.12869 28319300

[12] Tsou M-C, Özkaynak H, Beamer P, Dang W, Hsi H-C, Jiang C-B, et al. Mouthing activity data for children age 3 to <6 years old and fraction of hand area mouthed for children age <6 years old in Taiwan. J Expo Sci Environ Epidemiol. 2018;28(2):182–92. doi: 10.1038/jes.2016.87 28120832

[13] Chien L-C, Tsou M-C, Hsi H-C, Beamer P, Bradham K, Hseu Z-Y, et al. Soil ingestion rates for children under 3 years old in Taiwan. J Expo Sci Environ Epidemiol. 2017;27(1):33–40. doi: 10.1038/jes.2015.61 26443469

[14] Ngure FM, Humphrey JH, Mbuya MNN, Majo F, Mutasa K, Govha M, et al. Formative research on hygiene behaviors and geophagy among infants and young children and implications of exposure to fecal bacteria. Am J Trop Med Hyg. 2013;89(4):709–16. doi: 10.4269/ajtmh.12-0568 24002485 PMC3795101

[15] Bauza V, Ocharo RM, Nguyen TH, Guest JS. Soil Ingestion is Associated with Child Diarrhea in an Urban Slum of Nairobi, Kenya. Am J Trop Med Hyg. 2017;96(3):569–75. doi: 10.4269/ajtmh.16-0543 28093532 PMC5361529

[16] Jia T-W, Melville S, Utzinger J, King CH, Zhou X-N. Soil-transmitted helminth reinfection after drug treatment: a systematic review and meta-analysis. PLoS Negl Trop Dis. 2012;6(5):e1621. doi: 10.1371/journal.pntd.0001621 22590656 PMC3348161

[17] Gerber DJF, Dhakal S, Islam MN, Al Kawsar A, Khair MA, Rahman MM, et al. Distribution and treatment needs of soil-transmitted helminthiasis in Bangladesh: A Bayesian geostatistical analysis of 2017-2020 national survey data. PLoS Negl Trop Dis. 2023;17(11):e0011656. doi: 10.1371/journal.pntd.0011656 37930980 PMC10662736

[18] Capone D, Barker T, Cumming O, Flemister A, Geason R, Kim E, et al. Persistent Ascaris Transmission Is Possible in Urban Areas Even Where Sanitation Coverage Is High. Environ Sci Technol. 2022;56(22):15969–80. doi: 10.1021/acs.est.2c04667 36288473 PMC9671051

[19] Mertens A, Arnold BF, Benjamin-Chung J, Boehm AB, Brown J, Capone D, et al. Effects of water, sanitation, and hygiene interventions on detection of enteropathogens and host-specific faecal markers in the environment: a systematic review and individual participant data meta-analysis. Lancet Planet Health. 2023;7(3):e197–208. doi: 10.1016/S2542-5196(23)00028-1 36889861 PMC10009758

[20] Vaz Nery S, Traub RJ, McCarthy JS, Clarke NE, Amaral S, Llewellyn S, et al. WASH for WORMS: A Cluster-Randomized Controlled Trial of the Impact of a Community Integrated Water, Sanitation, and Hygiene and Deworming Intervention on Soil-Transmitted Helminth Infections. Am J Trop Med Hyg. 2019;100(3):750–61. doi: 10.4269/ajtmh.18-0705 30628573 PMC6402916

[21] Pickering AJ, Njenga SM, Steinbaum L, Swarthout J, Lin A, Arnold BF, et al. Effects of single and integrated water, sanitation, handwashing, and nutrition interventions on child soil-transmitted helminth and Giardia infections: A cluster-randomized controlled trial in rural Kenya. PLoS Med. 2019;16(6):e1002841. doi: 10.1371/journal.pmed.1002841 31242190 PMC6594579

[22] Kwong LH, Sen D, Islam S, Shahriar S, Benjamin-Chung J, Arnold BF, et al. Effect of sanitation improvements on soil-transmitted helminth eggs in courtyard soil from rural Bangladesh: Evidence from a cluster-randomized controlled trial. PLoS Negl Trop Dis. 2021;15(7):e0008815. doi: 10.1371/journal.pntd.0008815 34319986 PMC8351931

[23] Ercumen A, Benjamin-Chung J, Arnold BF, Lin A, Hubbard AE, Stewart C, et al. Effects of water, sanitation, handwashing and nutritional interventions on soil-transmitted helminth infections in young children: A cluster-randomized controlled trial in rural Bangladesh. PLoS Negl Trop Dis. 2019;13(5):e0007323. doi: 10.1371/journal.pntd.0007323 31050672 PMC6519840

[24] Benjamin-Chung J, Nazneen A, Halder AK, Haque R, Siddique A, Uddin MS, et al. The Interaction of Deworming, Improved Sanitation, and Household Flooring with Soil-Transmitted Helminth Infection in Rural Bangladesh. PLoS Negl Trop Dis. 2015;9(12):e0004256. doi: 10.1371/journal.pntd.0004256 26624994 PMC4666415

[25] Benjamin-Chung J, Crider YS, Mertens A, Ercumen A, Pickering AJ, Lin A, et al. Household finished flooring and soil-transmitted helminth and Giardia infections among children in rural Bangladesh and Kenya: a prospective cohort study. Lancet Glob Health. 2021;9(3):e301–8. doi: 10.1016/S2214-109X(20)30523-4 33607029 PMC7900607

[26] Cattaneo MD, Galiani S, Gertler PJ, Martinez S, Titiunik R. Housing, Health, and Happiness. American Economic Journal: Economic Policy. 2009;1(1):75–105. doi: 10.1257/pol.1.1.75

[27] Legge H, Pullan RL, Sartorius B. Improved household flooring is associated with lower odds of enteric and parasitic infections in low- and middle-income countries: A systematic review and meta-analysis. PLOS Glob Public Health. 2023;3(12):e0002631. doi: 10.1371/journal.pgph.0002631 38039279 PMC10691699

[28] Rahman M, Jahan F, Hanif S, Yeamin A, Shoab AK, Andrews JR, et al. Effects of household concrete floors on maternal and child health - the CRADLE trial: a randomised controlled trial protocol. medRxiv. 2024;:2024.07.26.24311076. doi: 10.1101/2024.07.26.24311076 40032381 PMC11877219

[29] Scrivener KL, John VM, Gartner EM. Eco-efficient cements: Potential economically viable solutions for a low-CO2 cement-based materials industry. Cement and Concrete Research. 2018;114:2–26. doi: 10.1016/j.cemconres.2018.03.015

[30] Ellis LD, Badel AF, Chiang ML, Park RJ-Y, Chiang Y-M. Toward electrochemical synthesis of cement-An electrolyzer-based process for decarbonating CaCO3 while producing useful gas streams. Proc Natl Acad Sci U S A. 2020;117(23):12584–91. doi: 10.1073/pnas.1821673116 31527245 PMC7293631

[31] Shah IH, Miller SA, Jiang D, Myers RJ. Cement substitution with secondary materials can reduce annual global CO2 emissions by up to 1.3 gigatons. Nat Commun. 2022;13(1):5758. doi: 10.1038/s41467-022-33289-7 36180443 PMC9525259

[32] Tosti L, van Zomeren A, Pels JR, Comans RNJ. Technical and environmental performance of lower carbon footprint cement mortars containing biomass fly ash as a secondary cementitious material. Resources, Conservation and Recycling. 2018;134:25–33. doi: 10.1016/j.resconrec.2018.03.004

[33] Venkateswara Rao A, Srinivasa Rao K. Effect of Fly Ash on Strength of Concrete. Circular Economy and Fly Ash Management. Springer Singapore. 2019. 125–34. doi: 10.1007/978-981-15-0014-5_9

[34] Babu KG, Nageswara Rao GS. Efficiency of fly ash in concrete. Cement and Concrete Composites. 1993;15(4):223–9. doi: 10.1016/0958-9465(93)90025-5

[35] Nath P, Sarker P. Effect of Fly Ash on the Durability Properties of High Strength Concrete. Procedia Engineering. 2011;14:1149–56. doi: 10.1016/j.proeng.2011.07.144

[36] Krithika J, Ramesh Kumar GB. Influence of fly ash on concrete – A systematic review. Materials Today: Proceedings. 2020;33:906–11. doi: 10.1016/j.matpr.2020.06.425

[37] Ogawa Y, Uji K, Ueno A, Kawai K. Contribution of fly ash to the strength development of mortars cured at different temperatures. Construction and Building Materials. 2021;276:122191. doi: 10.1016/j.conbuildmat.2020.122191

[38] Ravina D, Mehta PK. Properties of fresh concrete containing large amounts of fly ash. Cement and Concrete Research. 1986;16(2):227–38. doi: 10.1016/0008-8846(86)90139-0

[39] Rastogi A, Kumar Paul V. A Critical Review of the Potential for Fly Ash Utilisation in Construction-Specific Applications in India. EREM. 2020;76(2):65–75. doi: 10.5755/j01.erem.76.2.25166

[40] Islam MR, Hossain MI, Iqbal MB, Rahman M, Sarker A, Noor CA. Utilizing Fly Ash to Improve Subgrade Properties in Bangladesh. In: Airfield and Highway Pavements 2019, 2019. 522–30. doi: 10.1061/9780784482469.052

[41] Wang C, Liu K, Huang D, Chen Q, Tu M, Wu K, et al. Utilization of fly ash as building material admixture: Basic properties and heavy metal leaching. Case Studies in Construction Materials. 2022;17:e01422. doi: 10.1016/j.cscm.2022.e01422

[42] Fan C, Wang B, Ai H, Qi Y, Liu Z. A comparative study on solidification/stabilization characteristics of coal fly ash-based geopolymer and Portland cement on heavy metals in MSWI fly ash. Journal of Cleaner Production. 2021;319:128790. doi: 10.1016/j.jclepro.2021.128790

[43] Yu Q, Nagataki S, Lin J, Saeki T, Hisada M. The leachability of heavy metals in hardened fly ash cement and cement-solidified fly ash. Cement and Concrete Research. 2005;35(6):1056–63. doi: 10.1016/j.cemconres.2004.03.031

[44] Brownell SA, Nelson KL. Inactivation of single-celled Ascaris suum eggs by low-pressure UV radiation. Appl Environ Microbiol. 2006;72(3):2178–84. doi: 10.1128/AEM.72.3.2178-2184.2006 16517669 PMC1393194

[45] Nordin A, Nyberg K, Vinnerås B. Inactivation of Ascaris eggs in source-separated urine and feces by ammonia at ambient temperatures. Appl Environ Microbiol. 2009;75(3):662–7. doi: 10.1128/AEM.01250-08 19060175 PMC2632132

[46] Pecson BM, Nelson KL. Inactivation of Ascaris suum eggs by ammonia. Environ Sci Technol. 2005;39(20):7909–14. doi: 10.1021/es050659a 16295855

[47] American Concrete Institute. Standard Practice for Making and Curing Concrete Test Specimens in the Laboratory. 2020. https://www.astm.org/c0192_c0192m-18.html

[48] Cruz LM, Allanson M, Kwa B, Azizan A, Izurieta R. Morphological changes of Ascaris spp. eggs during their development outside the host. J Parasitol. 2012;98(1):63–8. doi: 10.1645/GE-2821.1 21801007

[49] Ravindran VB, Shahsavari E, Soni SK, Ball AS. Viability determination of Ascaris ova in raw wastewater: a comparative evaluation of culture-based, BacLight Live/Dead staining and PMA-qPCR methods. Water Sci Technol. 2019;80(5):817–26. doi: 10.2166/wst.2019.286 31746788

[50] Butkus MA, Hughes KT, Bowman DD, Liotta JL, Jenkins MB, Labare MP. Inactivation of Ascaris suum by short-chain fatty acids. Appl Environ Microbiol. 2011;77(1):363–6. doi: 10.1128/AEM.01675-10 21057018 PMC3019692

[51] Patil PP, Mutnuri S. Study of helminth eggs (Ascaris suum) inactivation by anaerobic digestion and electrochemical treatment. Gates Open Res. 2023;7:93. doi: 10.12688/gatesopenres.14573.1 39324031 PMC11422576

[52] Jeandron A, Ensink JHJ, Thamsborg SM, Dalsgaard A, Sengupta ME. A quantitative assessment method for Ascaris eggs on hands. PLoS One. 2014;9(5):e96731. doi: 10.1371/journal.pone.0096731 24802859 PMC4011755

[53] Faul F, Erdfelder E, Buchner A, Lang A-G. Statistical power analyses using G*Power 3.1: tests for correlation and regression analyses. Behav Res Methods. 2009;41(4):1149–60. doi: 10.3758/BRM.41.4.1149 19897823

[54] Cohen J. Statistical power analysis for the behavioral sciences. 2nd ed. Hillsdale, N.J: L. Erlbaum Associates. 1988.

[55] Bangladesh Meteorological Department. Temperature Data. Climate. https://live6.bmd.gov.bd/p/Monthly-Minimum-Temperature. 2014.

[56] Bangladesh Meteorological Department. Monthly Humidity Normal Data. Climate. https://live6.bmd.gov.bd/?/p/=Monthly-Humidity-Normal-Data. 2014.

[57] Greenspan L. Humidity fixed points of binary saturated aqueous solutions. J Res Natl BUR Stan SECT A. 1977;81A(1):89. doi: 10.6028/jres.081a.011

[58] Brown TW, Murphy JL, Akers P, Patrick M, Hill V, Mattioli M, et al. An environmental evaluation of urine-diverting dry toilets in Hiloweyn Camp, Dollo Ado, Ethiopia. Sci Total Environ. 2024;926:171838. doi: 10.1016/j.scitotenv.2024.171838 38518820 PMC13047511

[59] Harroff LA, Liotta JL, Bowman DD, Angenent LT. Current time-temperature relationships for thermal inactivation of Ascaris eggs at mesophilic temperatures are too conservative and may hamper development of simple, but effective sanitation. Water Res X. 2019;5:100036. doi: 10.1016/j.wroa.2019.100036 31535088 PMC6743028

[60] Fidjeland J, Nordin A, Vinnerås B. Inactivation of Ascaris eggs and Salmonella spp. in fecal sludge by treatment with urea and ammonia solution. Journal of Water, Sanitation and Hygiene for Development. 2016;6(3):465–73. doi: 10.2166/washdev.2016.017

[61] Arene FOI. Ascaris suum: Influence of embryonation temperature on the viability of the infective larva. Journal of Thermal Biology. 1986;11(1):9–15. doi: 10.1016/0306-4565(86)90011-2

[62] Schmitz B, Pearce-Walker J, Gerba C, Pepper I. A Method for Determining Ascaris Viability Based on Early-to-Late Stage In-Vitro Ova Development. JRST. 2016;13(4):275–86. doi: 10.12783/issn.1544-8053/13/4/5

[63] Steinbaum L, Kwong LH, Ercumen A, Negash MS, Lovely AJ, Njenga SM, et al. Detecting and enumerating soil-transmitted helminth eggs in soil: New method development and results from field testing in Kenya and Bangladesh. PLoS Negl Trop Dis. 2017;11(4):e0005522. doi: 10.1371/journal.pntd.0005522 28379956 PMC5393894

[64] Manser ND, Wald I, Ergas SJ, Izurieta R, Mihelcic JR. Assessing the fate of Ascaris suum ova during mesophilic anaerobic digestion. Environ Sci Technol. 2015;49(5):3128–35. doi: 10.1021/es505807a 25679819

[65] Senecal J, Nordin A, Vinnerås B. Fate of Ascaris at various pH, temperature and moisture levels. J Water Health. 2020;18(3):375–82. doi: 10.2166/wh.2020.264 32589622

[66] McKinley JW, Parzen RE, Mercado Guzmán Á. Ammonia inactivation of Ascaris ova in ecological compost by using urine and ash. Appl Environ Microbiol. 2012;78(15):5133–7. doi: 10.1128/AEM.00631-12 22582051 PMC3416402

[67] Grant MJ, Booth A. A typology of reviews: an analysis of 14 review types and associated methodologies. Health Info Libr J. 2009;26(2):91–108. doi: 10.1111/j.1471-1842.2009.00848.x 19490148

[68] Naidoo D, Foutch GL. The time-temperature relationship for the inactivation of Ascaris eggs. J Water Sanit Hyg Dev. 2017;8(1):123–6. doi: 10.2166/washdev.2017.102 33384866 PMC7734376

[69] Wardell R, Clements ACA, Lal A, Summers D, Llewellyn S, Campbell SJ, et al. An environmental assessment and risk map of Ascaris lumbricoides and Necator americanus distributions in Manufahi District, Timor-Leste. PLoS Negl Trop Dis. 2017;11(5):e0005565. doi: 10.1371/journal.pntd.0005565 28489889 PMC5440046

[70] Jensen PKM, Phuc PD, Konradsen F, Klank LT, Dalsgaard A. Survival of Ascaris eggs and hygienic quality of human excreta in Vietnamese composting latrines. Environ Health. 2009;8:57. doi: 10.1186/1476-069X-8-57 20003550 PMC2804663

[71] Fidjeland J, Nordin A, Pecson BM, Nelson KL, Vinnerås B. Modeling the inactivation of ascaris eggs as a function of ammonia concentration and temperature. Water Res. 2015;83:153–60. doi: 10.1016/j.watres.2015.06.030 26143272

[72] Ahmed AU, Sorensen DL. Kinetics of pathogen destruction during storage of dewatered biosolids. Water Environment Research. 1995;67(2):143–50. doi: 10.2175/106143095x131286

[73] Bachofer CS, Pahl G. Influence of Extended Temperature Treatments on Recovery of X-Irradiated Ascaris Eggs. Radiation Research. 1955;2(1):50. doi: 10.2307/3570230 14357581

[74] Fidjeland J, Magri ME, Jönsson H, Albihn A, Vinnerås B. The potential for self-sanitisation of faecal sludge by intrinsic ammonia. Water Res. 2013;47(16):6014–23. doi: 10.1016/j.watres.2013.07.024 23941983

[75] Ghiglietti R, Genchi C, Di Matteo L, Calcaterra E, Colombi A. Survival of Ascaris suum eggs in ammonia-treated wastewater sludges. Bioresource Technology. 1997;59(2–3):195–8. doi: 10.1016/s0960-8524(96)00147-2

[76] Katakam KK, Mejer H, Dalsgaard A, Kyvsgaard NC, Thamsborg SM. Survival of Ascaris suum and Ascaridia galli eggs in liquid manure at different ammonia concentrations and temperatures. Vet Parasitol. 2014;204(3–4):249–57. doi: 10.1016/j.vetpar.2014.05.017 24893691

[77] Katakam KK, Roepstorff A, Popovic O, Kyvsgaard NC, Thamsborg SM, Dalsgaard A. Viability of Ascaris suum eggs in stored raw and separated liquid slurry. Parasitology. 2013;140(3):378–84. doi: 10.1017/S0031182012001722 23127297

[78] Kato S, Fogarty E, Bowman D. Effect of aerobic and anaerobic digestion on the viability of Cryptosporidium parvum oocysts and Ascaris suum eggs. Int J Environ Health Res. 2003;13(2):169–79. doi: 10.1080/0960312031000098071 12745337

[79] Maya C, Ortiz M, Jiménez B. Viability of Ascaris and other helminth genera non larval eggs in different conditions of temperature, lime (pH) and humidity. Water Sci Technol. 2010;62(11):2616–24. doi: 10.2166/wst.2010.535 21099049

[80] Maya C, Torner-Morales FJ, Lucario ES, Hernández E, Jiménez B. Viability of six species of larval and non-larval helminth eggs for different conditions of temperature, pH and dryness. Water Res. 2012;46(15):4770–82. doi: 10.1016/j.watres.2012.06.014 22794801

[81] O’Donnell CJ, Meyer KB, Jones JV, Benton T, Kaneshiro ES, Nichols JS, et al. Survival of parasite eggs upon storage in sludge. Appl Environ Microbiol. 1984;48(3):618–25. doi: 10.1128/aem.48.3.618-625.1984 6541889 PMC241576

[82] Ogunyoku TA, Habebo F, Nelson KL. In-toilet disinfection of fresh fecal sludge with ammonia naturally present in excreta. Journal of Water, Sanitation and Hygiene for Development. 2015;6(1):104–14. doi: 10.2166/washdev.2015.233

[83] Pecson BM, Barrios JA, Jiménez BE, Nelson KL. The effects of temperature, pH, and ammonia concentration on the inactivation of Ascaris eggs in sewage sludge. Water Res. 2007;41(13):2893–902. doi: 10.1016/j.watres.2007.03.040 17524448

[84] Senecal J, Nordin A, Simha P, Vinnerås B. Hygiene aspect of treating human urine by alkaline dehydration. Water Res. 2018;144:474–81. doi: 10.1016/j.watres.2018.07.030 30075443

[85] Sossou SK, Sou/Dakoure M, Konate Y, Maiga AH, Funamizu N. Inactivation kinetics of indicator microorganisms during urea treatment for sanitizing finished compost from composting toilet. Journal of Water, Sanitation and Hygiene for Development. 2016;6(2):269–75. doi: 10.2166/washdev.2016.090

[86] Tharaldsen J, Helle O. Survival of Parasite Eggs in Livestock Slurry Utilized for Compost Heat. Acta Agriculturae Scandinavica. 1989;39(4):381–7. doi: 10.1080/00015128909438531

[87] Venglovsky J, Sasakova N, Gregova G, Papajova I, Toth F, Szaboova T. Devitalisation of pathogens in stored pig slurry and potential risk related to its application to agricultural soil. Environ Sci Pollut Res Int. 2018;25(22):21412–9. doi: 10.1007/s11356-017-0557-2 29090442 PMC6063326

[88] Yu Y-M, Cho Y-H, Youn Y-N, Quan JH, Choi I-W, Lee Y-H. Quantitative evaluation of viability- and apoptosis-related genes in Ascaris suum eggs under different culture-temperature conditions. Korean J Parasitol. 2012;50(3):243–7. doi: 10.3347/kjp.2012.50.3.243 22949754 PMC3428572

[89] Yu Y-M, Kim J-W, Na W-S, Youn Y-N, Choi I-W, Lee Y-H. Effects of some pesticides on development of Ascaris suum eggs. Korean J Parasitol. 2014;52(1):111–5. doi: 10.3347/kjp.2014.52.1.111 24623893 PMC3948987

[90] Hawksworth. The effect of temperature and relative humidity on the viability of Ascaris ova in urine diversion waste. Water Institute of Southern Africa. https://wisa.org.za/document/the-effect-of-temperature-and-relative-humidity-on-the-viability-of-ascaris-ova-in-urine-diversion-waste/. 2024 November 25.

[91] Vinnerås B, Björklund A, Jönsson H. Thermal composting of faecal matter as treatment and possible disinfection method--laboratory-scale and pilot-scale studies. Bioresour Technol. 2003;88(1):47–54. doi: 10.1016/s0960-8524(02)00268-7 12573563

[92] Ercumen A, Hossain MS, Tabassum T, Haque A, Rahman A, Rahman MH. Dirt floors and domestic animals are associated with soilborne exposure to antimicrobial resistant E. coli in rural Bangladeshi households. medRxiv. 2025. doi: Pending 10.1021/acs.est.5c10329 PMC12676737 41221823

[93] Naidoo D, Archer C, Louton B, Rodda N. Testing household disinfectants for the inactivation of helminth eggs on surfaces and in spills during pit latrine emptying. WSA. 2016;42(4):560. doi: 10.4314/wsa.v42i4.06

