# Supplementary material for: Investigating cement-based surfaces as a sustainable flooring solution to improve Ascaris Suum egg removal and inactivation in low-resource settings
Source: PLoS Negl Trop Dis. 2025 Oct 23;19(10):e0012919. doi: 10.1371/journal.pntd.0012919 (PMC12548874; doi:10.1371/journal.pntd.0012919)
Supplement: S1 Text — Table A: Cement-based tiles mix designs. Table B: Recovery percentages for experimental conditions. Table C: Regression results corrected and unadjusted data. Fig A: Ascaris suum egg development photos. Fig B: Natural Log reduction of Ascaris suum viable eggs by trial. Fig C: Number of Ascaris suum eggs applied. Fig D: Number of Ascaris suum eggs recovered. Fig E: Natural log reduction of eggs for corrected and unadjusted data. Fig F: Survival experiments Ascaris suum viable eggs by trial. Fig H: Survival experiments Ascaris suum developed eggs by trial. Fig G: Literature review of time to 99% inactivation of Ascaris species eggs. (DOCX) [file pntd.0012919.s001.docx]

**Supplemental Information**

**Investigating Cement-Based Surfaces as a Sustainable Flooring Solution to Improve *Ascaris* *suum* Egg Removal and Decay in Low-Resource Settings**

Claire E. Anderson ^1^, Suhi Hanif ^2^, Jason Hernandez ^1^, Yoshika Crider ^3^, Michael Lepech^1^, Sarah L. Billington^1^, Alexandria B. Boehm ^1^, Jade Benjamin-Chung ^2,3,4^

^1^ Department of Civil and Environmental Engineering, Stanford University, Stanford, CA, 94305

^2^ Department of Epidemiology and Population Health, Stanford University, Stanford, CA, 94305

^3^ King Center on Global Development, Stanford University, Stanford, CA, 94305

^4^ Chan Zuckerberg Biohub, San Francisco, CA, 94158

*Author to whom correspondence should be addressed

The supplemental material contains the following information, as referenced in the main article:

| **Complete Data Set**  Anderson, C. E., Hanif, S., Hernandez, J., Crider, Y., Lepech, M., Billington, S. L., Boehm, A. B., and Benjamin-Chung, J. (2025). Ascaris suum Survival and Removal Code and Complete Data Set. Version 2. Stanford Digital Repository. Available at https://purl.stanford.edu/rj162zg5361/version/3. https://doi.org/10.25740/rj162zg5361. |  |
| --- | --- |
| ***Contains Sheets***  Removal: Data from removal experiments  Survival MD / FD / FW / MW: Data from survival experiments  Stock Controls: Data from stock controls  Literature Review: Data from the literature review  Linear Regressions: Data from the linear regressions run by trial  Corrected Linear Regressions: Data from the linear regressions run by trial based on data adjusted for recovery differences over time |  |
| **Text** |  |
| Recovery correction calculation……….....………………………………………………………………………………… | S3 |
| **Tables** |  |
| Table A: Cement-based tiles mix designs..…………………………………………………………………………….. | S4 |
| Table B: Recovery percentages for experimental conditions…………………………………………………. | S5 |
| Table C: Regression results corrected and unadjusted data.…………………………………………………. | S6 |
| **Figures** |  |
| Fig A: *Ascaris suum* egg development photos.…………………………………………….…………………… | S7 |
| Fig B: Natural Log reduction of *Ascaris suum* viable eggs by trial.……………………………………… | S8 |
| Fig C: Number of *Ascaris suum* eggs applied..………………………………………………………………….. | S9 |
| Fig D: Number of *Ascaris suum* eggs recovered.…………………………………………………………….... | S10 |
| Fig E: Natural log reduction of eggs for corrected and unadjusted data.……………................ | S11 |
| Fig F: Survival experiments *Ascaris suum* viable eggs by trial.…………………………………………… | S12 |
| Fig H: Survival experiments *Ascaris suum* developed eggs by trial.…………………………………… | S13 |
| Fig G: Literature review of time to 99% inactivation of *Ascaris* speceis eggs……………………… | S14 |

**Recovery correction calculation**

To account for egg recovery efficiency, we applied a correction factor to Equation 3 from the main text. The corrected regression model is shown in Equation S1:

$ln(\frac{{Corrected\_N}_{V}}{{Corrected\_N}_{0}})=-kt+\beta_{0}$ **Equation S1**

where *t* is the time (days) on the cement-based surface, *k* is the first-order decay rate constant (days*^−^*^1^), and 𝛽_0_ is the y-intercept.

The corrected number of viable eggs after a time of *t* (days) on the cement-based surface, *Corrected_N_V_*, is given by:

$Corrected\_N_{V}=\frac{N_{V}}{R_{t}}$ **Equation S2**

where *N_V_* is the number of viable eggs after a time of *t* (days) and *Rₜ* represents the ratio of recovered eggs at time *t* to total eggs applied to the cement-based surface (Equation S3).

$R_{t}=\frac{Total eggs recovered at time=t}{Total eggs applied}$ **Equation S3**

The corrected initial number of viable eggs, *Corrected_N₀*, is given by:

$Corrected\_N_{0}=\frac{N_{0}}{R_{0}}$ **Equation S4**

where *N_o_* is the initial number of viable eggs and *R₀* represents the ratio of recovered eggs at time 0 to the total eggs applied to the cement-based surface (Equation S5).

$R_{0}=\frac{Total eggs recovered at time=0}{Total eggs applied}$ **Equation S5**

*R_0_* and *R_t_* values are shown as percentages in Table B. Data for $ln(\frac{{Corrected\_N}_{V}}{{Corrected\_N}_{0}})$ can be visualized in Fig e.

**Table A:** Mix design proportions for Ordinary Portland Cement (OPC) mortar, OPC fly ash mortar, and neat cement finishes.

| **Mix Design** | **Water-to-**  **Cementitious Material Ratio** | **Cement**  **(kg/m^3^)** | **Water**  **(kg/m^3^)** | **Fine Aggregate**  **(kg/m^3^)** | **Coarse Aggregate**  **(kg/m^3^)** | **Supplementary Cementitious Materials**  **(kg/m^3^)** |
| --- | --- | --- | --- | --- | --- | --- |
| OPC Mortar | 0.466 | 630 | 294 | 1200 | – | – |
| *Neat Cement Finish* | 0.466 | 1276 | 595 | – | – | – |
|  | | | | | | |
| OPC Fly Ash Mortar | 0.466 | 473 | 294 | 1200 | – | 158 |
| *Neat Cement Finish with Fly Ash* | 0.466 | 957 | 595 | – | – | 319 |

**Table B:** Recovery percentages for experimental conditions for each cement-based mix design, experimental condition, and time sampled. Also shown are the mean total number of applied eggs and the mean number of recovered eggs used to calculate the percentage of eggs recovered.

| **Mix Design and Environmental Condition** | **Time (Days)** | **Mean Number of Total Applied Eggs** | **Mean Number of Total Recovered Eggs** | **Percentage of Eggs Recovered** |
| --- | --- | --- | --- | --- |
| OPC  Mortar Mix  Wet Season  (34°C, 75% RH) | 0 | 523 | 176 | 34% |
|  | 2 | 523 | 177 | 34% |
|  | 14 | 523 | 271 | 52% |
|  | 28 | 523 | 264 | 51% |
| OPC  Mortar Mix  Dry Season  (15°C, 75% RH) | 0 | 523 | 100 | 19% |
|  | 2 | 523 | 130 | 25% |
|  | 14 | 523 | 165 | 32% |
|  | 28 | 523 | 163 | 31% |
| OPC Fly Ash  Mortar Mix  Wet Season  (34°C, 75% RH) | 0 | 498 | 115 | 23% |
|  | 2 | 498 | 177 | 36% |
|  | 14 | 498 | 195 | 39% |
|  | 28 | 498 | 177 | 36% |
| OPC Fly Ash  Mortar Mix  Dry Season  (15°C, 75% RH) | 0 | 498 | 120 | 24% |
|  | 2 | 498 | 180 | 36% |
|  | 14 | 498 | 186 | 37% |
|  | 28 | 498 | 204 | 41% |

**Table C:** Regression results for data corrected for recovery differences over time (shaded values) and unadjusted data. Unadjusted data is the same as that presented in Table 1 in the main text, but values are shown together for comparison. The table includes the first-order decay rate constants (*k*) and y-intercept values (𝛽_0_) for each cement-based mix design, experimental condition, and enumeration method used.

| **Mix Design** | **Environmental Condition** | **Enumeration Method** | ***k***  **(day^1^)** | **Corrected *k***  **(day^1^)** | ***k***  **p-value** | **Corrected**  ***k***  **p-value** | **𝛽_0_** | **Corrected**  **𝛽_0_** | **𝛽_0_**  **p-value** | **Corrected**  **𝛽_0_**  **p-value** | **R^2^** | **Corrected**  **R^2^** |
| --- | --- | --- | --- | --- | --- | --- | --- | --- | --- | --- | --- | --- |
| OPC Fly Ash Mortar | Wet Season  (34°C, 75% RH) | Viable | 0.15 | 0.166 | 2.3 x 10^-20^ | 1.3 x 10^-20^ | -0.24 | -0.27 | 0.10 | 9.6 x 10^-2^ | 0.83 | 0.84 |
|  |  | Developed | 0.060 | 0.076 | 1.1 x 10^-11^ | 8.8 x 10^-14^ | -0.23 | -0.26 | 0.03 | 3.0 x 10^-2^ | 0.61 | 0.67 |
|  | Dry Season  (15°C, 75% RH) | Viable | -1.1 x 10^-3^* | 0.014 | 0.82 | 1.3 x 10^-2^ | -0.14 | -0.29 | 0.07 | 1.4 x 10^-3^ | 1.0 x 10^-3^ | 0.12 |
|  |  | Developed | -7.9 x 10^-4^* | 0.014 | 0.87 | 9.4 x 10^-3^ | -0.12 | -0.27 | 0.10 | 2.0 x 10^-3^ | 5.6 x 10^-4^ | 0.13 |
| OPC Mortar | Wet Season  (34°C, 75% RH) | Viable | 0.13 | 0.144 | 2.9 x 10^-17^ | 2.0 x 10^-17^ | -0.32 | -0.55 | 0.03 | 1.3 x 10^-3^ | 0.81 | 0.79 |
|  |  | Developed | 0.066 | 0.077 | 1.1 x 10^-12^ | 9.6 x 10^-12^ | -0.21 | -0.44 | 0.07 | 9.6 x 10^-4^ | 0.63 | 0.64 |
|  | Dry Season  (15°C, 75% RH) | Viable | 1.4 x 10^-3^* | 0.015 | 0.80 | 1.6 x 10^-2^ | -0.10 | -0.29 | 0.25 | 2.5 x 10^-3^ | 1.3 x 10^-3^ | 0.11 |
|  |  | Developed | 1.5 x 10^-3^* | 0.015 | 0.78 | 1.5 x 10^-2^ | -0.09 | -0.29 | 0.27 | 2.8 x 10^-3^ | 1.2 x 10^-3^ | 0.11 |

* P-values for *k* indicate that the first-order decay rate constant is not significantly different from zero, and that therefore no inactivation was observed.


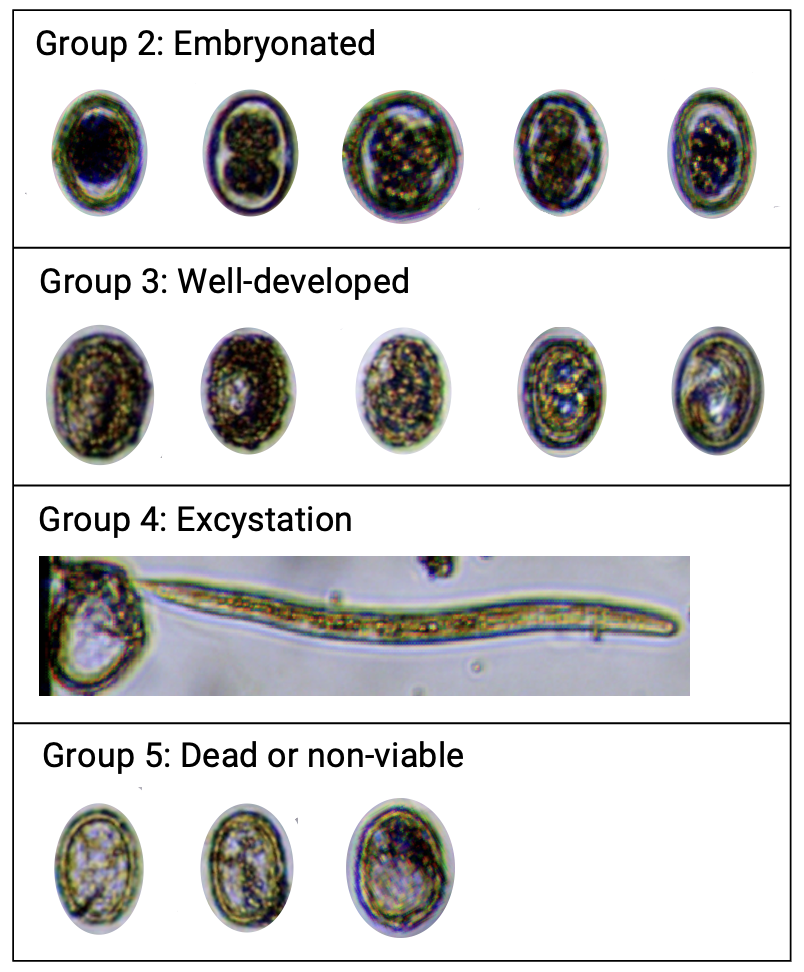


**Fig A:** *Ascaris suum* egg example development photos, separated by their development group. Photos were taken by author CEA at 10X magnification. Groups include (1) Unembryonated, stage 1 (not pictured); (2) Embryonated, stage 2 to 7; (3) Well-developed, stage 8 to 15; (4) Excystation, stage 16; and (5) Dead or non-viable. Not all stages in each group are pictured. Stages are based on Schmitz et al. 2016, as referenced in the main text.

**
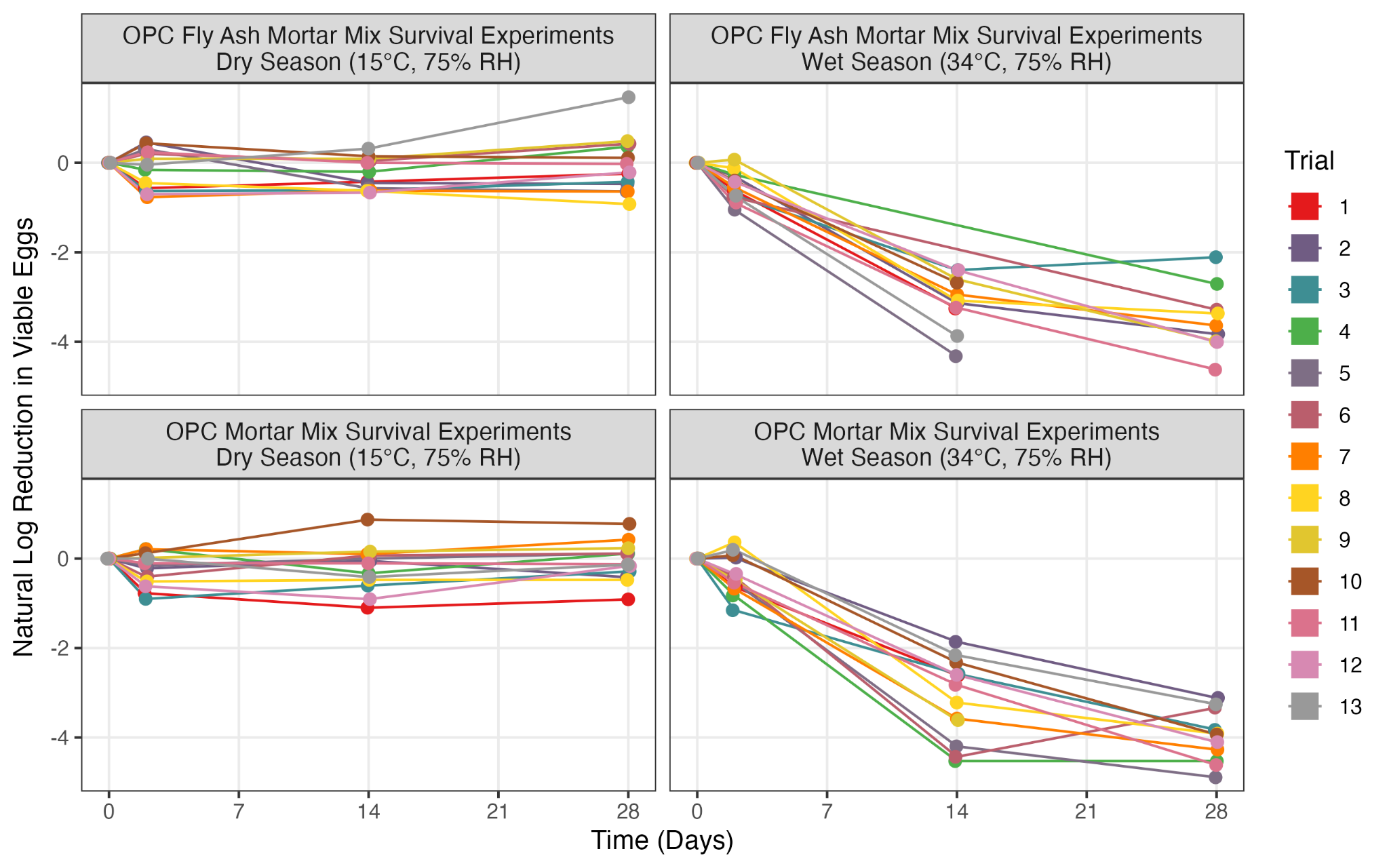
**

**Fig B:** Natural log reduction of *Ascaris suum* viable eggs (Equation 3 in the main text: $ln(\frac{N_{V}}{N_{0}})$) by trial for survival experiments. Samples were gathered on days 0, 2, 14, and 28 and are connected by lines. For some trials, no viable eggs were collected, so no data point is shown. Data is separated by season/temperature and tile type.

**
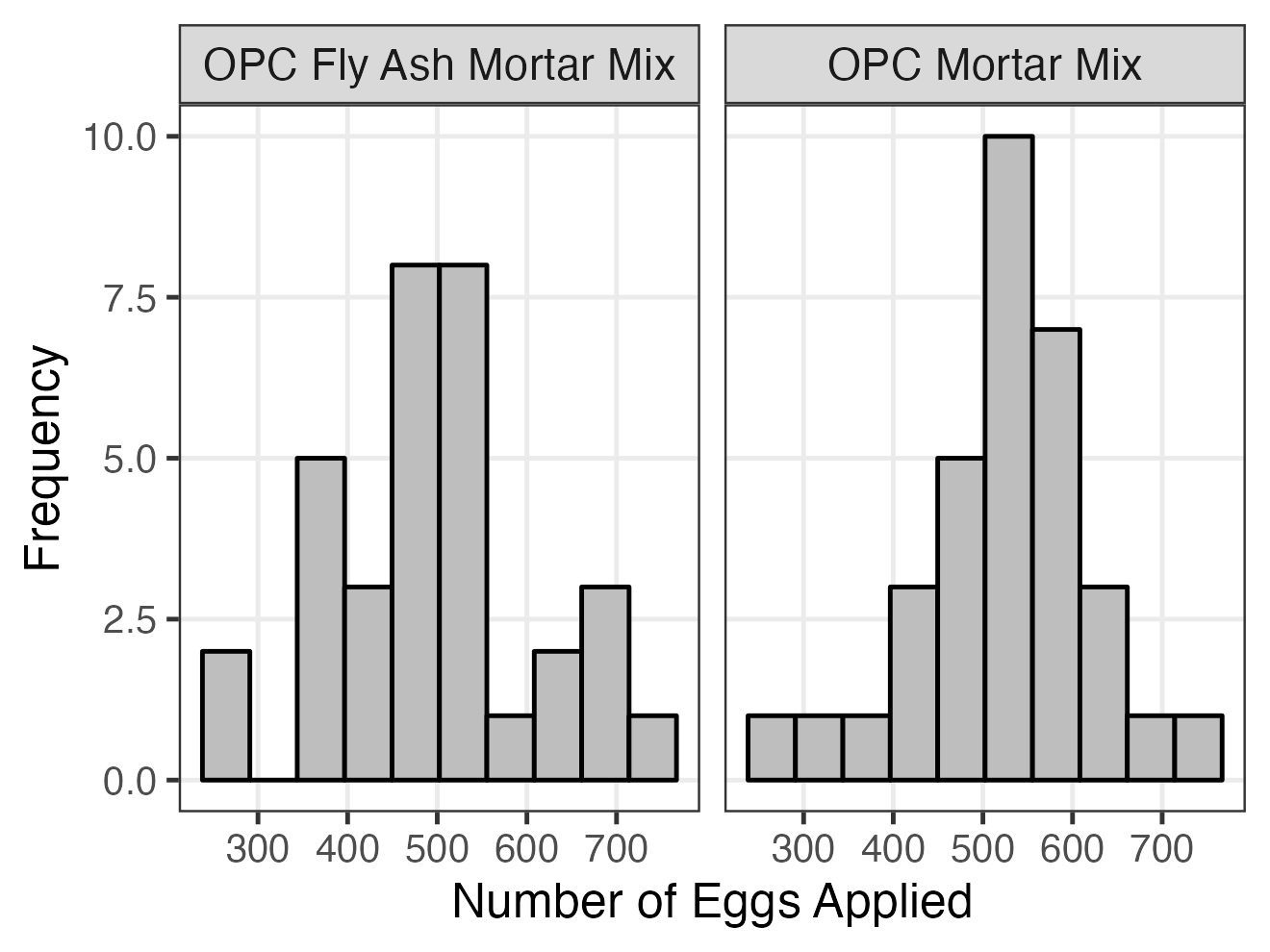
**

**Fig C:** Histogram of the number of total *Ascaris suum* eggs applied to the tiles. Data is separated by tile type.

**
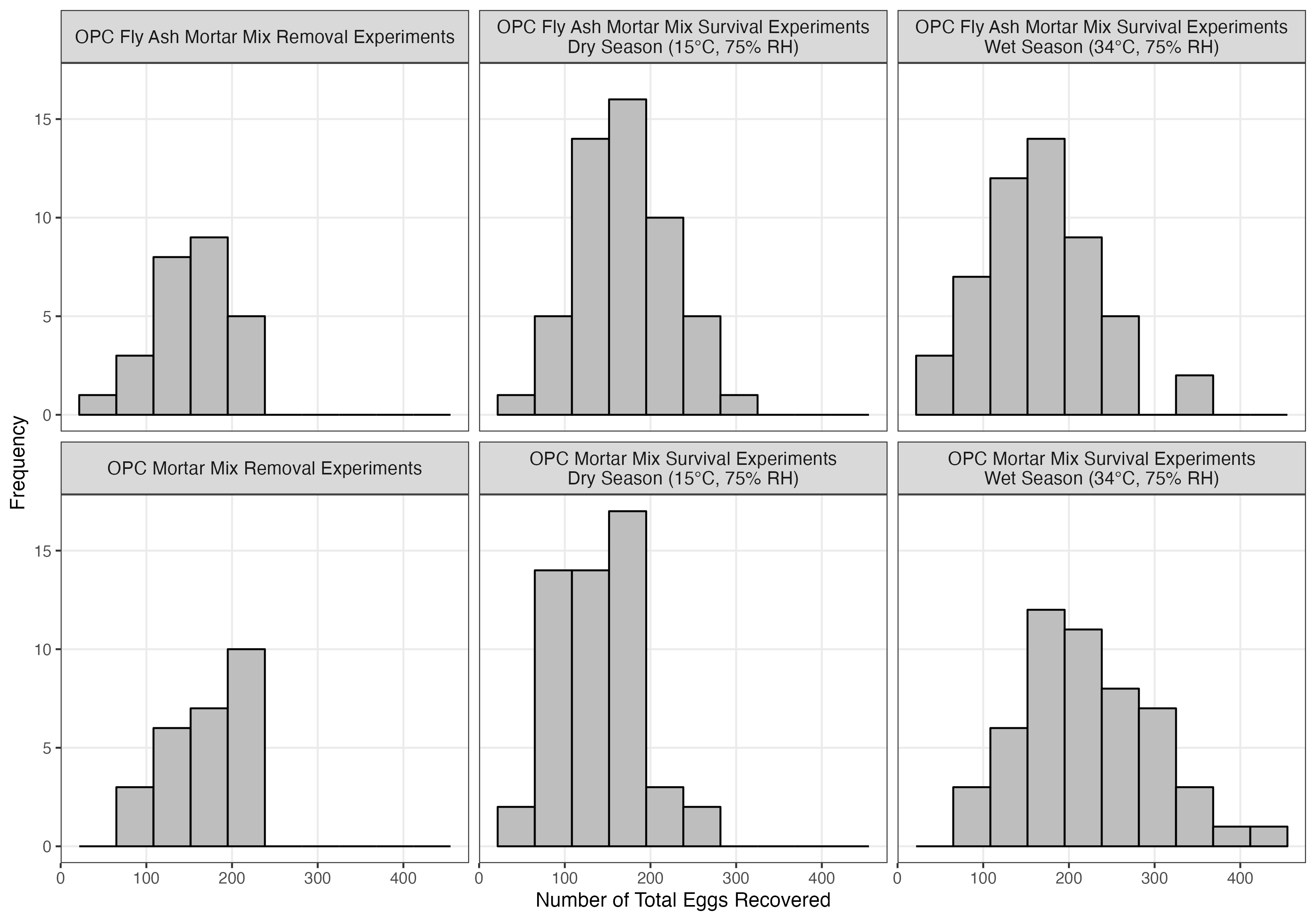
**

**Fig D:** Histogram of the number of total *Ascaris suum* eggs recovered from tiles. Data is separated by tile type for removal experiments and by season/temperature and tile type for survival experiments. Data is a combination of controls at time 0 for survival and removal experiments and recovery at days 2, 14, and 28 for survival experiments.

**
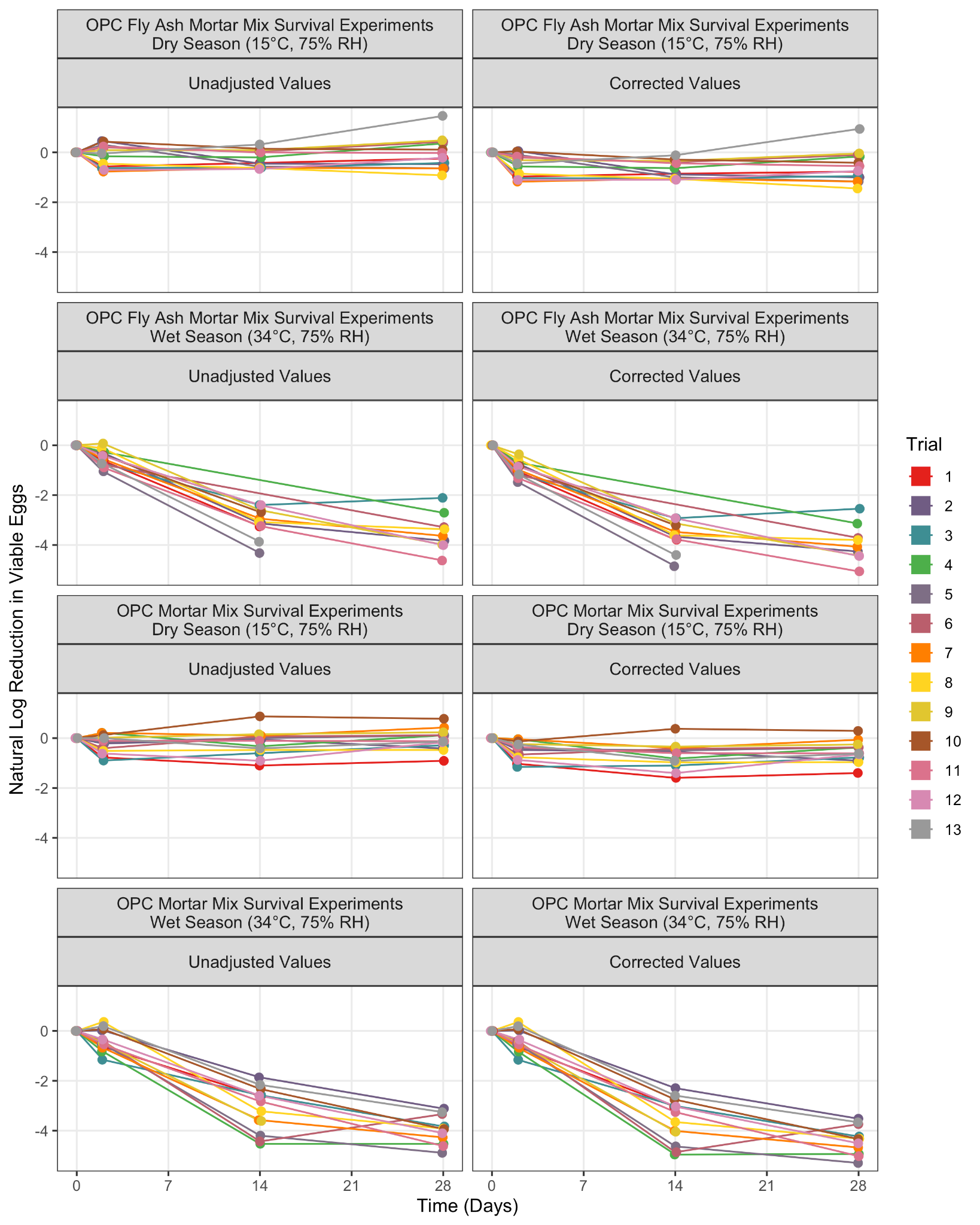
**

**Fig E:** Natural log reduction of *Ascaris suum* viable eggs (Equation 3 in the main text and Equation S1 in the supplemental information) by trial for survival experiments using data corrected for recovery differences over time and unadjusted data. Unadjusted data is the same as that presented in Fig B, but values are shown together for comparison. Samples were gathered on days 0, 2, 14, and 28 and are connected by lines. For some trials, no viable eggs were collected, so no data point is shown. Data is separated by season/temperature and tile type.

**
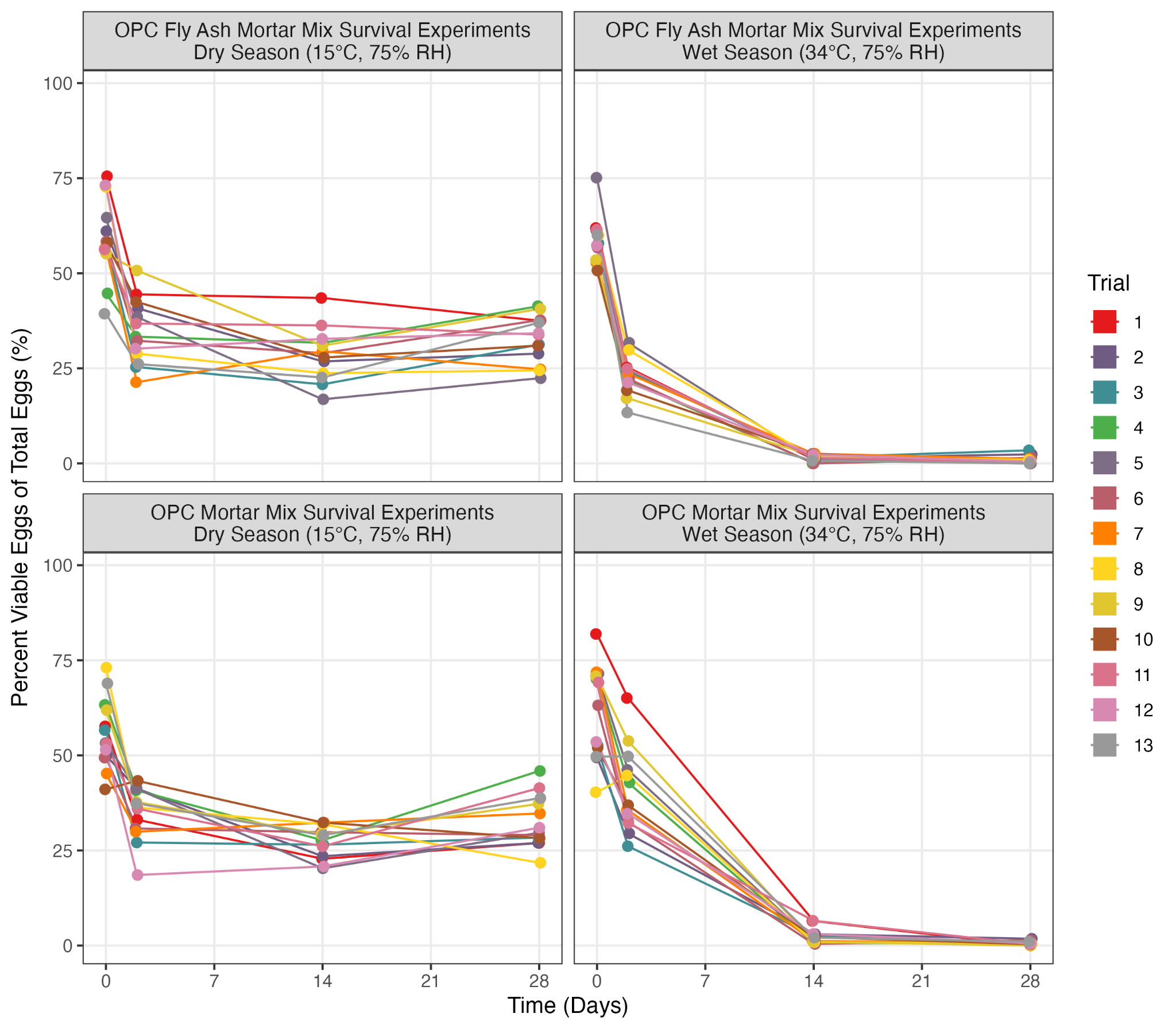
**

**Fig F:** Percent of *Ascaris suum* viable eggs of total eggs by trial for survival experiments. Samples were gathered on days 0, 2, 14, and 28 and are connected by lines. For some trials, no viable eggs were collected, so no data point is shown. Data is separated by season/temperature and tile type.

**
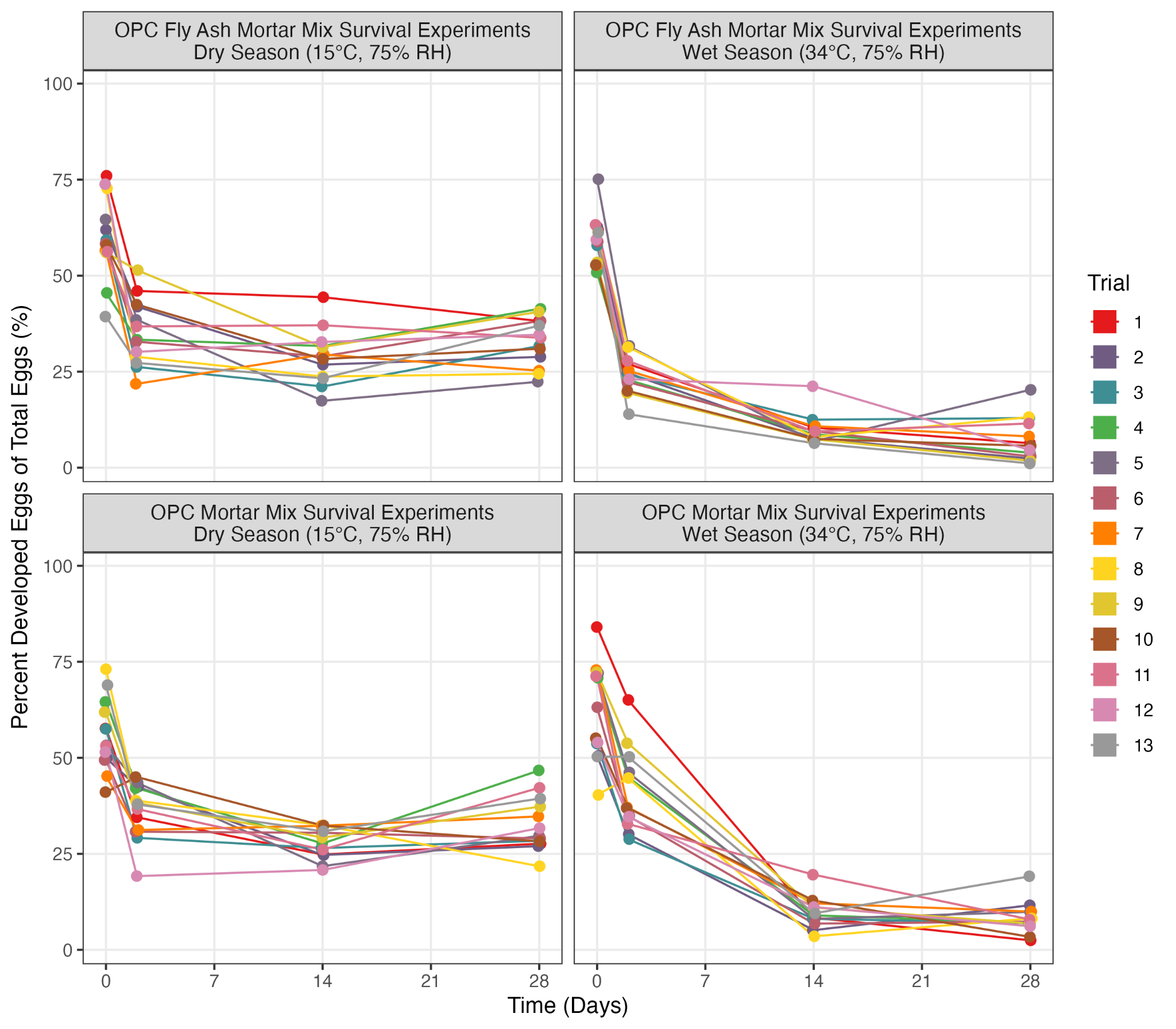
**

**Fig H:** Percent of *Ascaris suum* developed eggs of total eggs by trial for survival experiments. Samples were gathered on days 0, 2, 14, and 28 and are connected by lines. Data is separated by season/temperature and tile type.


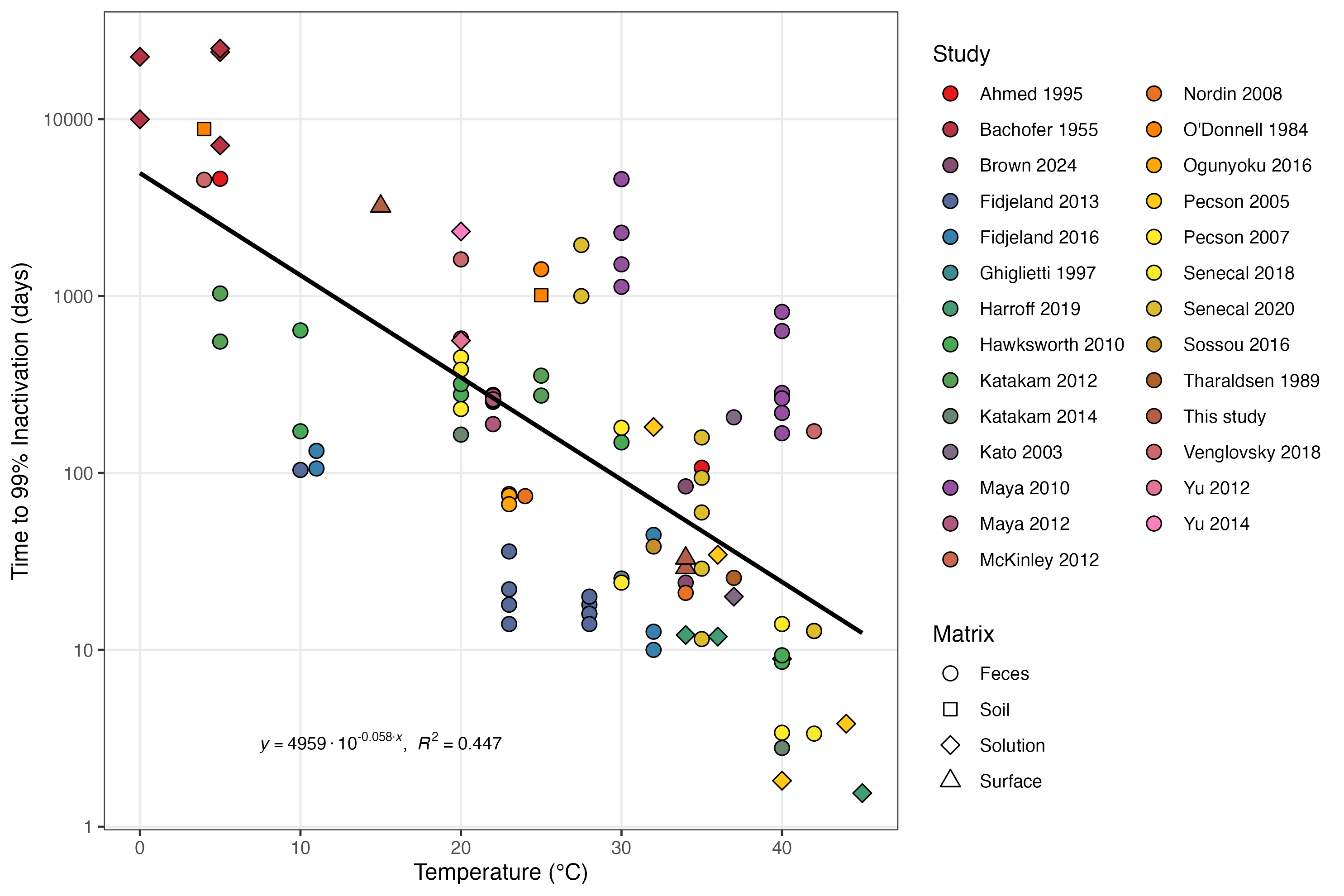


**Fig G:** Time to 99% inactivation of *Ascaris* species eggs at different temperatures from literature review results compiled from this study and previous studies. Data point color indicates the reference study and shape indicates the matrix of interest. A regression of the data, along with the equation and R^2^, are also plotted. Complete references and study information are within the “Complete Data Set” referenced in the SI table of contents.
